# Intervention Strategies for Prevention of Comorbid Depression Among Individuals With Type 2 Diabetes: A Scoping Review

**DOI:** 10.3389/fpubh.2019.00035

**Published:** 2019-03-05

**Authors:** Eva Guérin, Hamdi Jaafar, Lisa Amrani, Denis Prud'homme, Céline Aguer

**Affiliations:** ^1^Institut du Savoir Montfort-Recherche, Ottawa, ON, Canada; ^2^Department of Biochemistry, Microbiology, and Immunology, Faculty of Medicine, University of Ottawa, Ottawa, ON, Canada; ^3^Faculty of Health Sciences, School of Human Kinetics, University of Ottawa, Ottawa, ON, Canada

**Keywords:** type 2 diabetes, depressive disorder, prevention, comorbidity, diabetes education program

## Abstract

**Background:** Type 2 diabetes (T2D)-related depression has a significant impact on quality of life and leads to greater morbidity and mortality. Current educational and treatment programs for T2D rarely include a specific depression-prevention component, focusing largely on remediating depressive symptoms that have reached clinical levels.

**Objective:** Given the vast field of research on the association between T2D and depression, and the unknown status of prevention efforts for the latter, the goal of this scoping review was to conduct a synopsis of intervention strategies specifically targeting the prevention of depression among adults with T2D.

**Eligibility Criteria:** (1) participants aged 18 and over with T2D; (2) experimental and quasi-experimental designs (3) intervention strategies seeking to prevent the onset or worsening of (non-clinical) depressive symptoms; (4) a valid measure of depressive symptoms; (5) full-text articles available in English or French.

**Sources of Evidence:** Databases including Medline, PubMed, and SCOPUS were searched between 2000 and 2018 resulting in 4,219 potential articles.

**Charting Methods:** This review was conducted in-line with the current methodological framework for scoping reviews. Titles, abstract and full text articles were screened independently and in duplicate. A narrative analysis was conducted to synthesize study characteristics and the nature of intervention strategies and components.

**Results:** Twelve studies were identified with the primary aim of preventing the incidence of depressive symptoms or improving non-clinical depression levels. Individual and group-based approaches included educational interventions incorporating diabetes self-management, problem-solving, and resilience-focused approaches, emotion-targeted techniques as well as alternative interventions. Self-monitoring, home practices, and motivational interviewing were common elements.

**Conclusions:** This review lays the groundwork for future studies seeking to develop, validate, and improve prevention strategies targeting the diabetes-depression comorbidity. More studies over longer periods and with larger samples are needed to capture the effects of prevention efforts.

## Introduction

Diabetes mellitus, the seventh leading causes of death worldwide (1), is a metabolic disorder characterized by dysfunction in the production, secretion, or action of insulin, which results in a state of hyperglycaemia. Type 2 diabetes (T2D) represents 90% of the different forms of diabetes (2).

The societal and economic burden of diabetes is significant (3). In fact, the impact of T2D on quality of life, life expectancy, and premature morbidity and mortality is devastating (4). Premature deaths among patients with T2D are primarily attributable to complications of this disease, including heart disease, stroke, and peripheral neuropathy (5). Living with T2D and its corollaries can also have important mental health consequences that can affect the disease's course and treatment (6). Specifically, it is known that depression is a major comorbid disorder in individuals with T2D (7).

Studies have shown that the incidence of depression is up to three times greater among individuals with T2D than the general population (8), and one in four people with T2D develops clinically significant depression (9). Whereas, the term *diabetes distress* is used to define emotional distress stemming from the burden of living with a chronic disease (10), major depression is a clinical mood disorder characterized by persistent and severe feelings of sadness and/or disinterest (11). Other presenting symptoms include lack of sleep, loss or gain of appetite, lack of concentration, agitation, etc. as defined by the Diagnostic and Statistical Manual of Mental Disorders-V (DSM-V) criteria. Validated clinical tools are used to identify symptoms and establish a diagnosis of depression (12).

Diabetes-related depression has a significant impact on quality of life and significantly increases rates of morbidity and mortality (13, 14). In particular, depression influences health care behaviors among individuals with T2D, impacting their nutrition, treatment adherence, glycemic control, motivation, and productivity (15, 16). Likewise, several studies have shown that depression worsens T2D-related complications, primarily stemming from poorer glycemic control and an impaired ability to manage diabetes-related comorbidities (e.g., hypertension) in general (17). Moreover, there is evidence of an interactive effect of the two disorders, such that decrements in health go beyond their additive harm (18). Therefore, it is not surprising that healthcare expenses are significantly higher among individuals with T2D and depression (13). Therefore, it is critical to identify strategies that will prevent the development and progression of depression among people with T2D.

For individuals with T2D, available treatment plans usually target cardiometabolic factors (e.g., glycaemia, blood pressure, and lipids) and diabetes management. There is an abundance of clinical programs aimed at glycemic control as well as exercise and nutritional programs targeting weight loss and improved insulin action (19). Such interventions primarily focus on underlying biological factors of T2D rather than psychological factors and depression specifically. Nonetheless, greater knowledge of the incidence and severity of depression in individual with T2D has propelled research on depression management and treatment options, though results have been mixed (9). Among the most commonly cited interventions are those combining psychotherapy with pharmacotherapy in addition to standard T2D management guidelines (20, 21). Conventional intervention elements include education about both illnesses, healthy lifestyle behaviors, and the acquisition of problem solving skills, and their effectiveness is more pronounced for T2D individuals with mild or moderate depressive symptoms (22, 23).

However, the volume, availability, and content of educational interventions delivered to individuals with T2D that include a specific depression-*prevention* component is unknown. To our knowledge, interventions to date have focused primarily on remediating individuals with T2D once depressive symptoms have reached clinical levels (24, 25). Given the aforementioned costs and burden of this comorbidity and considering high rates of non-adherence and failure of T2D treatments among major depression suffers (13–17), it seems paramount to properly target risk factors for depression in individuals with T2D in order to aid in its prevention. It is therefore critical to understand the current state of knowledge and identify fruitful avenues for future prevention studies.

The objective of this study was to identify available intervention strategies to prevent the onset or the worsening of depressive symptoms among individuals with T2D. Given the vast field of work on T2D and its associated mental health complications, and the unknown status of research on preventing depression, we carried out a scoping review with the goal of gathering and mapping scientific information. We conducted a narrative synopsis of existing intervention strategies that can be used to target the prevention of depression among individuals with T2D. In this reviewer, we refer to depressive symptoms as the different signs and manifestations that characterize depression rather than a clinical (DSM-V) diagnosis of major depressive disorder (i.e., five or more symptoms, >2 weeks, causing significant impairment).

## Methods

### Study Design and Protocol

The goal of a scoping review is to explore a complex and broad theme of research and to provide a global synthesis of content and/or results on a particular topic. A scoping review is preferred when there may be deficiencies or uncertainties in the volume of literature on a topic or heterogeneity in regards to various facets of the research, including population, measurement instruments, and interventions (26). Scoping reviews identify and describe existing studies on a particular subject by considering a variety of designs and without systematically extracting and appraising results in relation to methodological quality. In *scoping* reviews, researchers are not expected to conduct a meta-analysis of the available data. For this review, a protocol was prepared (available on request) in accordance with guidelines put forward by the Joanna Briggs Institute (27, 28). The conduct of this scoping review was consistent with the Preferred Reporting Items for Systematic Reviews and Meta-Analysis (PRISMA) extension for Scoping Reviews (PRISMA-ScR) which have since been recently released (29).

### Eligibility Criteria

The inclusion criteria were follows: (1) participants aged 18 and over with diagnosed (or otherwise confirmed or established) T2D; (2) experimental design such as randomized controlled trials as well as quasi-experimental designs (including pre-post schemes), cohort studies, case control studies, and mixed-method studies; (3) any form of prevention approach or intervention strategy including educational programs or other seeking to prevent the onset of depression or prevent the worsening of (non-clinical) depressive symptoms; (4) a valid measure of depressive symptoms, for example, the Beck Depression Inventory (BDI) (30), and the Center for Epidemiological Studies Depression Scale (CES-D) (31); (5) full-text articles available in English or French.

Exclusion criteria included: (1) participants <18 years old and other diabetes types (e.g., type, gestational); (2) presence of clinical complications of diabetes (including, but not limited to heart disease, nephropathy, retinopathy, stroke), (3) diagnosis of major depressive disorder or other clinical mood disorder; (4) interventions to *treat* depression (e.g., pharmacological, psychological, behavioral, etc.) where the primary objective was remediating an existing (i.e., clinical/diagnosed or otherwise elevated) depressive state rather than preventing onset or worsening of depressive symptoms; (5) interventions that target multiple chronic diseases (e.g., T2D and heart disease); (6) correlational and observational studies focused on prevalence of depression and or T2D or on predictive factors; (7) systematic reviews, meta-analyses, dissertations, or conference presentation abstracts^1^; (8) sole focus on screening or diagnosis of depression (including instrument validation studies).

### Information Sources and Search Strategy

Pertinent articles were identified by searching several databases, including Medline, Embase, Cochrane Database of Systematic Reviews, PsycINFO, PubMed, and Scopus. A search strategy was developed and tested using a combination of terms (Supplemental File 1). A preliminary exploration of the keywords “type 2 diabetes,” “depression,” and “prevention” in PubMed resulted in only 6 manuscripts published between 1986 and 1999. The number of publications increased significantly after 2000, reflecting a growing interest in this area post-2000. The search was thus limited to publication dates between January 2000 and May 2018. Articles reporting secondary analyses of data were included. The final search strategy was deemed satisfactory following independent screening by two evaluators (EG, LA) of the first 100 excluded articles to confirm warranted removal.

### Source Selection (Screening)

Results of the database search were uploaded to Distiller Systematic Review (DSR) software to facilitate the process of article screening and selection as well as data extraction between the three reviewers (EG, LA, HJ). Screening forms were developed in DSR for each level of screening. These were pilot tested and refined accordingly by the evaluators.

Independently and in duplicate, the three evaluators screened titles for relevance. Titles deemed relevant by one or both evaluators were retained for abstract screening. Similarly, the evaluators subsequently examined the list of abstracts to identify those deemed worthy of full article screening. In the case of disagreement during abstract screening, discrepancies were discussed until agreement was reached regarding inclusion. Lastly, the full-texts of the remaining articles were surveyed for final inclusion. As required, an impartial third evaluator (CA) provided their advice regarding the article's selection.

### Data Items and Charting Process

For all studies included in the final review, data were extracted conforming to the PICO framework with the following variables:

#### Population

Sample size, location of study, sex or gender, ethnicity, age, and if available HbA1c levels, body mass index (BMI), and duration of T2D (months or years).

#### Intervention

Intervention target, intervention type and modality (e.g., mindfulness) and/or intervention approach (e.g., cognitive behavioral therapy), delivery (i.e., group vs. individual), context (e.g., community, hospital, home, etc.), dose (when applicable, i.e., length of sessions in minutes), frequency (i.e., number of sessions per week), duration (i.e., weeks or months).

#### Comparator

(if applicable) number of comparators and type(s) of comparator(s) (e.g., control group, active control, distinct treatment, etc.).

#### Outcome

Primary depression measure (instrument), list of all outcomes evaluated in the study (i.e., primary and secondary outcomes reported in published article); time-points of assessment. In regards to depression, as the goal of this study was not to systematically evaluate intervention effects quantitatively, a general synopsis of impact was extracted. Specifically, the following information was pulled for each intervention study as available: (a) statistical change in depression over time from baseline (i.e., in terms of symptom severity or level of depression) and/or (b) statistical change in the prevalence of depression among participants over time.

As the goal of this scoping review was to identify and examine studies in which the main aim was preventing the onset or worsening of depressive symptoms, included studies were divided as follows: (1) depressive symptoms or levels as the primary or sole outcome; (2) depressive symptoms or levels as a secondary or unspecified intervention measure or outcome.

### Synthesis of Results

The reviewed studies were first described according to study and intervention characteristics (Table 1), participant characteristics (Table 2), and intervention effects on depression (Table 3). We conducted a narrative, descriptive synthesis of contextual delivery factors and active intervention ingredients. In a scoping review, experts recommend a qualitative content analysis be conducted in light of the emerging body of evidence on the topic as well as variability in study designs and participant samples. In our study, we consider the similarities, differences, and implications of the included intervention strategies, including their design and implementation.

**Table 1:** Study and intervention characteristics for 12 included studies.

**Study and location**	**Design**	**Intervention and type**	**Group vs. individual**	**Comparators and type**	**Intervention duration, dose, and frequency**
Abazarian et al. (32)/ Iran	Quasi-experimental (case control study)/2 groups	Teaching problem solving and decision making courses	Unclear	1 group/Nothing (no instruction)	8 sessions
Cohn et al. (33)/ USA	Randomized intervention design/2 groups	DAHLIA: Developing Affective HeaLth to Improve Adherence/Self-paced, online intervention	Individual	1 group/Emotion-reporting waitlist control	Variable (5 weeks total); Average of 5 internet visits/week
Davies et al. (34)/ UK	Multicentre cluster RCT in primary care/13 sites/2 groups	Structured group education programme based on three psychological and learning theories/Community setting (routine care)	Group	1 group/Usual care	360 min total; 1 full day or 2 $\frac{1}{2}$ days
Dennick et al. (35)/ USA	Feasibility RCT/2 groups	Written emotional disclosure (WED)	Individual	1 group/Neutral writing	12 weeks/20 min; 3 days/week
Fisher et al. (36)/ USA	Cluster randomized clinical RCT/2 groups	Collaborative, structured self-monitoring of blood glucose (SMBG)	Individual	1 group/Active control group (ACG) (enhanced usual care with quarterly diabetes focused physician visits, free blood glucose meters/strips)	48 weeks (12 months); Variable dose (time required to register 7 points on SMBG profile for 3 consecutive days); 4 visits
Mansour et al. (37)/ Egypt	Pre-post experimental design/2 groups	Group-based counseling and education program (nursing intervention)	Group	1 group/No intervention	2 weeks; Variable dose in min; 2 sessions/week
Pearson et al. (39)/ Tasmania	RCT/2 groups	Self-directed intervention of mindfulness practice (IMP) using audio CD	Individual/at home	1 group/control (blank CD, usual care from clinic)	8 weeks/daily
Putiri et al. (40)/ USA	3-arm RCT (clinical trial)/3 groups	YRMQ (Yi Ren Medical Qigong)/Qigong therapy (traditional Chinese medicine) delivered by a certified instructor	Group + individual	2 groups/1. Active comparator (progressive resistance training; PRT); 2. Standard Care	12 weeks; YRMQ & PRT: 60 min + 2 × 30 min group sessions; 1 group session + 2 individual practice sessions (30 min)/week
Sardar et al. (41)/ Iran	RCT/2 groups	Aerobic exercise training	Unclear	1 group/ No exercise activities	8 weeks; 45–60 min; 3x/week
Steinhardt et al. (42)/ USA	Pre-post design pilot study/1 group	Diabetes Coaching Program adapted for African Americans/2 courses: resilience intervention delivered by health education professor; nutrition education related to diabetes, delivered by PhD candidate	Group	None	8 weeks; Courses: 120 min/week; 4 weeks Group meetings: 90 min/2 weeks
Wagner et al. (43)/ USA	RCT/2 groups	Manualized and culturally-based Community Health Worker (CHW)—delivered Stress Management (SM) intervention with Diabetes Education (DE)	Group	1 group/ DE only	8 sessions over 8–10 weeks (DE + SM)/2.5 h or 1 session (DE)
Zagarins et al. (44)/ USA	Pre-post design/1 group	Diabetes self-management education intervention (DSME)/Diabetes knowledge and skills training; fosters behavior change for self-management behaviors	Group	None	24 weeks/1 × 60 min session followed by 3 × 30 min sessions (at 1, 3, 6 months)

*RCT, Randomized controlled trial; FU, Follow-up; min, Minutes; YRMQ, Yi Ren Medical Qigong*.

**Table 2:** Participant characteristics for 12 included studies (results provided for the full sample unless otherwise specified).

**References**	**Sample size total/per group**	**Age (years) (mean ±SD, median or range)**	**Sex (% male)**	**Ethnicity (%)**	**Duration of T2D, years or months**	**% HbA1c (mean ±SD)**	**BMI in kg/m^**2**^) (mean ± SD)**
Abazarian et al. (32)	30/Interv: 15	Interv: 44 Control: 47	NA	NA	NA	NA	NA
Cohn et al. (33)	42/Interv: 25	Median: 54	49.1%	36% Caucasian, 23% African American, 19% Asian or Asian American, 8% non-White Hispanic, 15% other	NA	NA	NA
Davies et al. (34)	824/Interv: 437	Interv: 59 (28–87) Control: 60 (29–87)	Interv: 53% Control: 57%	94% White European	≤12 weeks	Interv: 8.3 ± 2.2/Control: 7.9 ± 2.0	Interv: 32.3 ± 6.1 Control: 32.4 ± 6.5
Dennick et al. (35)	41/Interv: 23	65.6 ± 9.9 (41–84)	61%	98% White British; 2% Black Irish	7 years (84.0 ± 74.2 months)	7.0 ± 1.0	30.4 ± 6.4
Fisher et al. (36)	483/Interv: 256	55.8 ± 10.7	53.2%	31.1% African American, 63.1% Caucasian, 9.8% other	7.6 ± 6.1 years	8.9 ± 1.2	35.1 ± 7.3
Mansour et al. (37)	120/Interv: 60	≥40 (45% 50–55)	41.7%	NA	≥3 years (51.7% 5–10 years)	NA	NA
Pearson et al. (39)	67/Interv: 31	Interv: 57.5 ± 12.9 Control: 61.1 ± 11.8	Interv: 39% Control: 67%	NA	Interv: 11.7 ± 7.8 Control: 14.7 ± 9.1	Interv: 8.71 ± 1.5 Control: 7.96 ± 1.4	Interv: 36.2 ± 10.3 Control: 34.2 ± 9.2
Putiri et al. (40)	32/YRMQ: 11, Active control: 11, Standard care: 10	56.3 ± 8.1	41%	NA	NA	YRMQ: 8.8 ± 1.1% Active control: 8.6 ± 1.2% Standard care: 7.9 ± 0.8%	31.8 ± 6.0
Sardar et al. (41)	53/Interv: 27	Interv: 44.93 ± 5.35 Control: 45.56 ± 5.41	100%	NA	Interv: 5.2 ± 2.4 years Control: 5.4 ± 3.40 years	Interv: 7.66 ± 1.31%/Control: 7.18 ± 1.01	NA
Steinhardt et al. (42)	12	54.83 (43–66)	50%	100% African American	NA	6.94 ±1.70	32.83 ±5.36
Wagner et al. (43)	107/DE+SM: 61	DE+SM: 60.0 ± 11.2 DE: 60.8 ± 12.1	DE+SM: 28% DE: 26%	Self-identified Latino or Hispanic	≤6 months	NA	NA
Zagarins et al. (44)	234	55.7 ± 10.2 (31–80)	41%	84.1% White, 12.1% Latino	8.2 ± 7.0 years	8.9 ± 1.2 %	34.5 ± 6.4

*Interv, Intervention Group; NA, Not Available (or not applicable); YRMQ, Yi Ren Medical Qigong; MBCT, Mindfulness-based Cognitive Therapy; DE, Diabetes Education; SM, Self-Management*.

**Table 3:** Depression measurement, outcomes and results for 12 included studies.

**References**	**Measure of depression****^a^**	**Depression measure timepoints**	**Baseline depression levels (mean ±SD)**	**Effect of intervention on depression**	**Other outcomes**
Abazarian et al. (32)	BDI (21-item)	Baseline/Post-intervention	Interv: 35.10 Control: 34.30	Sig. decrease in mean at endpoint for interv. (23.10) vs. control (33.64)/Sig. difference between groups in pre-post change (p < 0.0001)	Anxiety
Cohn et al. (33)	CES-D	Baseline/Endpoint- (~57 and 63 days post-baseline for interv vs. control, respectively)	Interv: 16.9 ± 11.6 Control: 17.1 ± 15.4	Sig. greater reduction in depression for interv. vs. control (−4.3 vs. +0.6) (β = –.21, p = 0.05)	Attitudes; perceived stress; diabetes self-efficacy; diabetes distress; health behaviors (e.g., exercise)
Davies et al. (34)	HADS	Baseline/4, 8, 12 months FU	16% women and 8% men with score ≥8 on HADS/Median Interv: 2 Median Control: 3	Depression scores lower in interv. vs. control at all time points/Sig. difference between groups at 12 month FU (p = 0.032)	HbA1c; weight; serum lipids and cholesterol; quality of life; emotional distress; beliefs in illness; smoking; physical activity
Dennick et al. (35)	CES-D (exclusion of scores ≥16)	Baseline/12 weeks	Interv: 7.0 ± 1.0 Control: 6.4 ± 1.2	Sig. worsening of depressive symptoms for interv. vs. control group (p = 0.006)/Potentially clinically sig. difference at follow-up (9.9 vs. 5.1)	Feasibility; fidelity; diabetes distress; perceived health; self-care behaviors
Fisher et al. (36)	PHQ-8	Baseline/12 months	Interv: 6.54 ± 6.0 Control: 5.85 ± 5.4	Sig. reduction at 12 months in both groups (p < 0.0001)/For patients with moderate symptoms (PHQ-8≥) at baseline, sig. greater reduction for interv. vs. control (p = 0.04)	Diabetes distress; HbA1c
Mansour et al. (37)	The Zung self-rating depression scale (38)	Baseline/3 months	Depression severity for Interv: 27% none, 58.4% mild, 14.6% severe Control: 10% none, 57.7% mild, 32.5% severe	Interv: increase % of no depression and decrease in % mild and severe (t = 2.506, = 0.016) Control: decrease in % no depression and % severe and increase in % mild (t = 2.506, p = 0.016).	None listed
Pearson et al. (39)	DASS-21	Baseline/8 weeks, 12 weeks	Interv: 10.8 ± 8.9 Control: 7.3 ± 8.7	Sig. group × time interaction for depression (p = 0.02) with overall reduction in depression score of 4.1 units	Anxiety; stress; diabetes distress; diabetes self-care; HbA1c; blood pressure
Putiri et al. (40)	BDI (21-item)	Baseline/12 weeks	YRMQ: 7.4 ± 8.8 Active control: 5.2 ± 2.6 Standard care: 5.0 ± 3.1	YRMQ: lowered scores by 14.3% (non-sig.) Active control: sig. lowered scores by 50% (p < 0.03) Standard care: No sig. change	Perceived stress
Sardar et al. (41)	Mental Health Questionnaire (GHQ-28)	Baseline/8 weeks	Interv: 7.18 ± 0.52/Control: 7.29 ± 0.58/	No sig. difference in change between groups (p = 0.657)	Subscales of GHQ-28: physical symptoms, anxiety and insomnia, social functioning
Steinhardt et al. (42)	CES-D	Baseline/6 months, 8 months	Interv: 11.57 ± 7.08	No sig. change post-intervention	Resilience, diabetes empowerment, perceived stress, diabetes self-management, problem-focused coping, distal outcomes (e.g., BMI, HbA1c)
Wagner et al. (43)	PHQ-8 (Spanish)	Baseline/Post-treatment	DE+SM: 6.7 ± 5.9/DE: 5.3 ± 4.4	Sig. improvement in symptoms of depression for DE + SM (medium effect size)	Anxiety symptoms; diabetes distress; diabetes self-care; health status; glycemic control; cortisol
Zagarins et al. (44)	HADS	Baseline/6 months	Interv: 16.3 ± 1.8	Trend toward a decrease (Δ = −0.2, SD = 8.4) in depressive symptoms (p = 0.08)	None listed

*Interv, Intervention Group; Sig, Significant; FU, Follow-up; RCT, Randomized controlled trial; FU, Follow-up; SD, Standard Deviation; CES-D, Center for Epidemiology Studies Short Depression Scale; PHQ, Patient Health Questionnaire; HADS, Hospital anxiety depression scale; BDI, Beck Depression Inventory; Depression, Anxiety, and Stress Scale-21; BMI, Body Mass Index; YRMQ, Yi Ren Medical Qigong*.

a *Item version specified when available*.

### Risk of Bias

Consistent with scoping review recommendations and the Joanna Briggs Institute manual (28, 29), an appraisal of the quality of included sources and an assessment of risk of bias were not conducted (27).

## Results

### Selection of Sources and Characteristics

**Figure 1:**
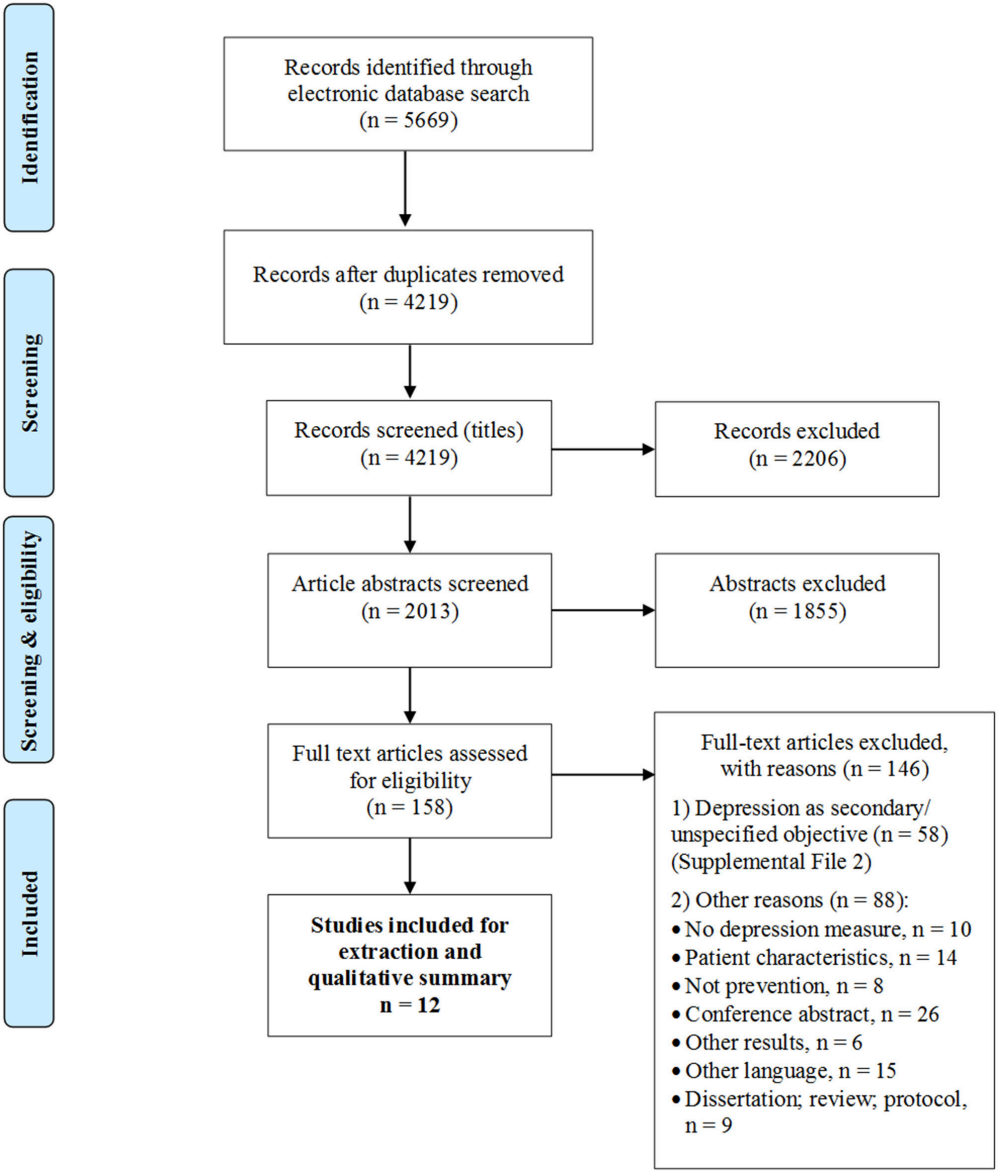
PRISMA flow diagram of included studies.

The initial search identified 4,219 studies for triage after duplicate removal (Figure 1). After inspection of the titles, the reduced list consisted of 2013 entries for abstract screening. Filtering of the abstracts resulted in 158 articles deemed relevant for full-text screening. The product of the full-text examination consisted in 12 eligible articles that focused specifically on preventing depression or improving (non-clinical) depressive symptoms as a primary objective, and 56 studies in which depression was considered a secondary aim (i.e., a non-priority outcome or not explicitly defined). For the purposes of this study, we present a detailed synopsis of the 12 intervention studies that focused on preventing depression (Tables 1–3). A tabular overview of the secondary studies is available as a Supplementary Table S1.

As shown in Table 3, the most common measure of depression was the CES-D (31). Four studies (33, 35, 36, 39) were individual-based while five (34, 37, 42–44) used group formats and one study (40) reported a combination of individual and group approaches. Two studies (32, 41) did not specify the delivery format and at least one study (33) exploited an online delivery. Eight of the 12 studies (33–36, 39–41, 43) used a variant of a randomized design while three studies (37, 42, 44) followed a pre-post scheme and one (32) used a quasi-experimental design. The duration of each intervention was variable, ranging from a few hours [e.g., 360 min over 2 days (34)] to multiple practice sessions (individual, group) several times per week for 12 weeks [e.g., Putiri et al. (40)].

Based on the findings presented in Table 3, a significant positive effect on levels of depression was reported in six of the 12 studies (32–34, 36, 39, 43).

In the sections that follow, key intervention components and delivery strategies are described for the 12 included studies. We grouped the interventions on a spectrum from structured programs (i.e., focus on T2D management and education), to problem-solving and emotion-based approaches to alternative and exercise-based intervention strategies.

### Synthesis of Results

#### Structured Programs: T2D Management and Education

Fisher et al. (36) tested a collaborative, structured self-monitoring of blood glucose (SMBG) intervention wherein participants also recorded meal sizes and calorie composition. Participants received instructions about how to identify problematic glycemic patterns. They recorded their SMBG profile during three consecutive days prior to scheduled study visits. During visits, forms were reviewed with a physician and suggestions for changes to exercise, nutrition, and medication were made. Given positive results of the intervention on depressive symptoms, particularly among participants with more elevated depression scores, the authors concluded that this type of collaborative approach between patient and physician could help patients deal with the emotional strain of managing their T2D. In itself, self-monitoring of glucose is a strongly advocated strategy among health professionals for facilitating treatment adherence (45). The observational element is also thought to improve patient experiences and encourage more positive attitudes toward T2D, thereby increasing disease and treatment satisfaction (46). This may in turn prevent the development of negative mood patterns in individuals with T2D.

Focused educational programs may be used in conjunction with specific diabetes management. In a cluster randomized RCT, Davies et al. (34) tested the Diabetes Motivation Strengthening (DESMOND) intervention for ongoing and newly diagnosed individuals with T2D. The program was grounded in a philosophy of patient empowerment and based on several learning theories (47). The design of the program was particularly suitable for preventing depression given its implementation within 12 weeks of T2D novo diagnosis. Key process elements included its delivery in the community by registered health professionals and its integration into routine care. A non-didactic approach was favored to elicit learning with an emphasis on activating participants to move toward specific, achievable goals in self-management (34). The content of the learning curriculum was broad, with a focus on risk and lifestyle factors. Overall, participants who received the intervention experienced less depression at 12 months than those in the standard education control condition.

Zagarins et al. (44) also examined a multifaceted Diabetes Self-Management Education (DSME) intervention. Participants received four sessions over a 6-month period by trained instructors, two of whom also incorporated motivational interviewing, thus strengthening the patient-clinician relationship and empowering patients to improve their health. This collaborative element is similar to the approach used by Fisher et al. (36). Although levels of depression did not diminish significantly from baseline, Zagarins et al. (44) suggested that this type of intervention could be more effective for preventing depression if implemented over a longer term. Depressive symptoms were not specifically addressed in the DSME-based intervention despite their importance as an outcome of the study, therefore an intensified focus on depression may be required.

#### Problem Solving and Resilience Building Approaches

Developing skills to help individuals manage their T2D is a common goal among educational programs in diabetes care and this often includes problem-solving. Researchers in Iran (32) tested an intervention that was based on a model stipulating that solving psychosocial problems can mediate the impact of stressful events on well-being (48). While intervention strategies by Davies et al. (34) and Fisher et al. (36) employed more standard approaches that help individuals manage their T2D by focusing on the disease itself, the approach from Abazarian et al. (32) had a distinct emphasis on wellness. In particular, an instruction-based intervention was delivered via eight sessions focused on recognizing problems, on key aspects of decision-making such as values and feelings, and on problem solving skills such as evaluating resources. The positive influence of the intervention on depression levels of participants at endpoint provided evidence that improving daily decision-making can help participants improve their well-being in the face of problems related to managing T2D.

Effective problem solving can also help individuals with T2D build resilience. Steinhardt et al. (42) pilot tested a 4-week Diabetes Coaching Program (DCP), adapted for African Americans, which combined a resilience intervention with nutrition education. The content of *Transforming Lives Through Resilience Education* was consistent with a resilience model based on previous work on the topic [e.g., Carver (49)]. Examples of curriculum components in weeks 1 and 3 were problem- and emotion-focused coping strategies and an empowering interpretation model. With nutrition education, the goal was to provide realistic, practical suggestions for improving diabetes care (e.g., reading food labels). Bi-weekly support-group follow-up meetings were devoted to problem solving, reviewing course content, and providing social support. Noted benefits in distal outcomes like HbA1c levels were not, however accompanied by decreases in depressive symptoms post-intervention. Akin to Zagarins et al. (44), it was suggested that a longer duration might be required to influence key psychological processes.

Another culturally sensitive intervention was delivered by Wagner et al. (43) over a period of 8–10 weeks, and with favorable results. In the Community Health Workers Assisting Latinos Manage Stress and Diabetes (CALMS-D) trial, diabetes education was paired with stress management training. The educational piece covered basic information about managing diabetes (e.g., nutrition, medication, glucose monitoring). Most notably, the core components of the manualized intervention were group psychoeducational skills training and physiological relaxation skills training. At each session, learning objectives were framed around a culturally relevant analogy or story. In session and at-home relaxation activities (e.g., via CD) were used in combination. Improvements in symptoms of depression with the intervention were significant, suggesting that interventions focused explicitly on managing stress and the psychological symptoms of diabetes are justified.

#### Emotion-Based Approaches and Positive Psychology

A subset of interventions focused on individuals' emotional health in lieu of diabetes-specific content. Mansour et al. (37) showed equivocal support for a counseling intervention focused directly on preventing clinical depression, though they offered very little description of the nature of the counseling method itself. The researchers were attentive to creating an optimal environment in which to develop of an effective therapeutic relationship. Specifically, their intervention rested on principles of trust and attentive listening and on therapist qualities such as demonstrating interest, concern, and friendliness, which naturally require a higher level of training and competencies from counselors and resources (time).

Intensive counseling interventions stand in stark contrast with the approach taken by Dennick et al. (35). The team implemented Written Emotional Disclosure (WED), a theoretically grounded, self-administered low-intensity technique that has the potential to respond to low-level psychological needs. Backed by the premise that WED is comparatively inexpensive and more widely available for widespread dissemination, participants were instructed to engage in 20 min writing sessions at home in private on 3 days over 1 week. Participants were incited to write their thoughts and feelings regarding any stressful experience over the last month or a current, non-diabetes concern. Results showed a worsening effect for the WED group, notably depressive symptoms were significantly more severe at 3-month follow-up. Coupled with feasibility issues, there were important limitations in the potential effectiveness of the WED intervention for use in primary care in its current form. The authors proposed a better evaluation of the appropriateness of WED based on individual characteristics and readiness, to which can be added the need for more proactive psychological skills training.

In this regard, Developing Affective HeaLth to Improve Adherence (DAHLIA) was an online, self-paced intervention that taught positive affect skills in order to improve daily positive emotions and adaptation as well as general coping abilities and well-being (33). Grounded in the Stress and Coping Theory (50, 51) and the Broaden-and-Build theory of positive emotion (52), targeted skills included positive reappraisal, setting attainable goals, performing acts of kindness, and mindfulness. Participants learned to practice one or more of these skills every day via weekly lessons and home assignments. Participants were encouraged to keep a learning journal and to apply acquired skills to manage their T2D and in other life domains. A mindfulness practice (i.e., 10 min reflection and mindful breathing) was promoted to harness an awareness of positive events. Results showed a significant reduction in symptoms of depression with the intervention, regardless of baseline levels (33). The authors reported good adherence and argued that an online format offers a low-cost alternative intervention to prevent depression among individuals with T2D who have more limited options for face-to-face care.

#### Alternative Approaches and Exercise

Mindfulness can be used as an adjunct strategy in multicomponent interventions like DAHLIA or it may be a worthy approach in and of itself. As demonstrated by Cohn et al. (33), an advantage of mindfulness is its potential to be self-directed. In an effectiveness study among individuals with T2D who demonstrated a need for psychological support, Pearson and colleagues (39) examined a novel delivery approach for mindfulness practice. Over an 8 week period, patients used an audio compact disc (CD) to engage in a daily 30 min mindfulness intervention. The CD was developed by a trainer with over 25 years of mindfulness practice. Participants in the intervention group showed an overall reduction in depression scores at 12 weeks.

Mindfulness is one of several alternative practices that teach awareness regarding the interplay between movement, breath and thoughts. Putiri et al. (40) compared Yi Ren Medical Qigong (YRMQ) to progressive resistance training and standard care in adults with T2D. Qigong is a traditional Chinese practice designed to maintain health and cultivate spiritual well-being. Exercises are performed with a heighted sense of feeling and focus which cultivates self-awareness of internal energy conditions. Although weekly YRMQ delivered by a certified instructor lowered participants' BDI scores by 14.3%, the effect was not significant. Since developing a regular Qigong practice may take time, an intervention period longer than 12 weeks may be necessary to achieve success. Interestingly, participants in the progressive resistance-training physical activity (PA) group showed significant reductions in depression.

Conversely, the results of another study that targeted aerobic PA behavior specifically were not consistent in regards to depression. In Sardar et al. (41), participants engaged in an aerobic exercise-training program (45–60 min on an ergocycle at 60–70% of maximum heart rate, three times per week for 8 weeks). Unfortunately, a detailed description of the context in which the exercise training was delivered and what strategies were used to ensure adherence throughout were lacking. Although the intervention was consistent with recommendations for aerobic training in diabetes, it did not result in significant changes in depression in this all-male sample. It is likely that the duration, intensity, and type of exercise training may need to be considered, particularly given that enjoyment of the activity may have particular relevance in regards to depression.

Nonetheless, various forms of PA and lifestyle change have been the focus of many intervention studies to date, including those considered in the current review in which depression was presented as a secondary or auxiliary outcome (53–59). Descriptive information and main findings of these studies are available as a Supplementary Table S1. These tables can be consulted to extract learning points and relevant strategies for developing future intervention studies.

## Discussion

### Summary of Evidence

Over four thousand titles met our initial search strategy but only 12 studies satisfied our inclusion criteria. We determined that the volume of research studies that have developed and tested intervention strategies focusing specifically on preventing depression in individuals with T2D is limited. Interestingly, in the initial exclusion stages of our review we identified several studies relating to the *treatment* of depression. Specifically, over 10% of the consulted abstracts and over a dozen full-texts were excluded based on a treatment approach for diagnosed or confirmed clinical depression. This suggests that research to date has overwhelmingly emphasized alleviating depressive symptoms once they develop rather than preventing the development or progression of depressive symptoms.

Still, our narrative review allowed us to hone in on the general success, limitations, and key elements of preventative approaches that have been tested, namely educational interventions incorporating diabetes self-management, problem-solving and resilience-focused approaches, emotion-targeted techniques as well as alternative interventions.

Overall, six studies with a primary focus on depression showed a significant positive effect while six studies showed inconsistent or non-significant findings. However, given that study quality was not assessed (e.g., using GRADE or a similar tool) and that we acknowledge variations in study design and reporting of results, the effectiveness of the interventions should be interpreted with caution. Nonetheless, we were able to extract specific components that can be integrated in revised interventions and tested over longer periods, in larger samples and including both sex.

### Key Intervention Components and Future Studies

Self-management education and support (SMES) has a long history in diabetes care. Consistent with National Standards for SMES (60) and studies we presented, interventions can take on many forms and include components of nutritional and lifestyle counseling, safe medication practices, monitoring of glucose and other parameters, and the development of personal strategies to deal with psychosocial problems. In the larger context of chronic disease, programs that include a psychotherapeutic component (behavioral or psychosocial) tend to demonstrate better results as they allow patients to modify negative perspectives about their illness, which can be a principal factor in developing comorbid mental disorders such as depression (61, 62). One review showed a large combined effect of psychotherapy and self-management education in treating depression among individuals with T2D (21). In the CALMS-D trial (43), a specific focus on stress management in facing diabetes and on building psychoeducational skills yielded superior improvements compared to education only. Thus, even if levels of personal responsibility for T2D increase with an intervention, participants may feel better equipped to manage it (34). Therefore, we suggest that incorporating a defined psychological element to patient education might be valuable for preventing the development or the exacerbation of depressive symptoms.

Moreover, studies have shown that T2D self-management programs that are adapted to the age, language, and culture of individuals are more effective compared to generic programs (63–65). This may be particularly true in the context of addressing depression given different risk factors between racial and ethnic groups as well as cultural values and expectations about mental disorders (66). Cultural adaptation can take many forms that extent beyond translation, including the integration of culturally salient values and an understanding of stigma related to living with depression. Apart from Steinhardt et al. (42), Wagner et al. (43), and a few secondary studies targeting Latinos specifically [e.g., Castillo et al. (67), Wang et al. (68)], few studies seemed to explicitly integrate or describe cultural adaptations. A unique characteristic of T2D management interventions is that they are typically patient-centered and adapted to patient needs, which should include evaluating multiple parameters such as beliefs and attitudes (60). Therefore, future studies could strive to consider the ethno-linguistic background and diversity of targeted participants and the appropriateness of activities and messages.

The capacity to adapt or individualize an intervention may vary as a function of delivery. Online interventions can offer flexible options that address individual preferences and needs (33, 69). In addition, online interventions can be delivered anonymously, at lower cost, and at convenient locations and times, which may increase efficacy (33, 70). While at least six secondary studies used a computer-based or online delivery (57, 70–73) and another four tested mobile phone applications (74–77), only one of the primary studies reported using a web-based format (33). Specifically, the DAHLIA positive affect intervention lead to lower levels of depression compared to an emotion-reporting waitlist control condition (33). Other mediums for self-directed or home-based interventions such as the audio for a mindfulness practice (39) have also shown promise, although issues regarding how to measure and address compliance and adherence may compromise their potential (70).

In addition, since elements of interaction, interpersonal dynamics, and peer learning are lacking from screen/web based approaches, commitment to the intervention and thus effectiveness may wane over time. In the context of depression, a meta-analysis demonstrated the potential for web-based interventions to significantly improve well-being outcomes in individuals with T2D, but not depression specifically (69). Yet, given RCT evidence that web-based self-help intervention can reduce the incidence of major depressive disorder over 12 months in participants not affected by T2D (78), further testing is warranted.

On the other hand, research has shown that group-based (in-person) diabetes education programs are particularly effective for improving clinical, lifestyle, and psychosocial outcomes in individuals with T2D (79–81). Our findings in the context of reducing depressive symptoms strengthen the efficacy of group approaches. Allowing participants to share their thoughts and insights may be particularly pertinent to problem-solving interventions, where skills, advice and strategies can be learned and integrated among group participants with similar experiences (32, 42, 43). It is noteworthy that some interventions herein emphasized not only problem solving and coping skills in relation to diabetes, but also general psychosocial preparedness, competencies, and resilience. Thus, when considering the mental health risks that accompany the demands of a disease like T2D, it may be beneficial to integrate an awareness of an individual's life and emotional experiences beyond the disease itself (32).

In particular, we saw the emergence of interventions that were more holistic in nature. Two studies (33, 39) suggested a distinct mindfulness component to complement their programs, with the DAHLIA intervention (33) also targeting patients' general cognitions and emotions. The practice of mindfulness helps people take a nonjudgmental and observing stance on their thoughts, leading to less worry and rumination, which are important correlates of depression (82). Regarding implementation, mindfulness is a unique approach in that unlike mainstream or other diabetes-orientated interventions, it can be delivered as an adjunct therapy by non-specialists and can be a cheaper and more accessible long-term alternative (83). It has shown promise for preventing symptoms of depression in patients with type 1 and 2 diabetes (84). Given that the incidence of T2D and depression are increasing at alarming rates, further research is needed to develop low-cost approaches and alternative means of delivery that will be available to a more diverse population of individuals with T2D.

## Limitations

While the strengths of this review include a comprehensive search of multiple databases, a rigorous screening process for inclusion, and an explicit focus on prevention, the findings should be interpreted within the scope of a few limitations. Firstly, this review does not provide a quantitative summary of effect size. Thus, we cannot objectively discuss efficacy or compare efficacy across the different intervention delivery formats. Likewise, we did not assess moderators of intervention effects, thus we cannot conclude for whom (e.g., age group) and under what conditions (i.e., latency of T2D diagnosis) preventive programs are most effective. Similarly, common mechanisms linking diminished depressive symptoms to improvements in glycemic control with treatment (or other disease indicators) were not examined and could be explored in future studies. Secondly, although we excluded studies that did not utilize a valid measure of depressive symptoms, there was variability in the choice of instruments across studies, which could have led to differences in terms of effects and participant selection (i.e., symptom severity cut-offs). Thirdly, the inclusion of a study was based on the specification of depression as a primary outcome and it is likely that some studies were excluded, or depression was deemed secondary, based on semantics. Lastly, the timeline for outcome measurement in regards to prevention was not objectively defined (i.e., 6 months), thus we do not know whether the changes observed in some studies were fleeting improvements or sustained prevention.

## Conclusions

More research is needed in the preluding or early stages of depression development in individuals with T2D such that the exacerbation of depressive symptoms can be avoided. Thwarting rates of major depression in this population has important implications in terms of treatment success, recovery, and healthcare costs (85, 86). Our findings suggest that incorporating a psychological component can be of practical benefit in comprehensive T2D educational approaches; specific attention to an individual's general mental state while cultivating positivity and well-being is also promising. Future studies will help us understand how to tailor preventive efforts to sub-group of individuals with T2D as well as how and when to deliver specific program components.

## Author Contributions

EG, HJ, LA, DP, and CA defined the search strategy. EG, HJ, and LA did the article screening, selection, and data extraction. EG conducted the synthesis and drafted the narrative summary. HJ, DP, and CA revised the summary and manuscript critically for important intellectual content.

### Conflict of Interest Statement

The authors declare that the research was conducted in the absence of any commercial or financial relationships that could be construed as a potential conflict of interest.

## Footnotes

^1^As the objective of this scoping review was not to evaluate efficacy but rather the intervention components, we excluded reviews from our analyses, although the reference lists were checked for relevant studies.
