# Supplementary material for: Intervention Strategies for Prevention of Comorbid Depression Among Individuals With Type 2 Diabetes: A Scoping Review
Source: Front Public Health. 2019 Mar 5;7:35. doi: 10.3389/fpubh.2019.00035 (PMC6411710; doi:10.3389/fpubh.2019.00035)
Supplement: Supplementary file 1 [file Table_1.docx]

Table S1 : Descriptive information and main findings for studies in which depression was unspecified or presented as a secondary or auxiliary outcome.

| **Study** | | **Design and sample characteristics** | | | | **Intervention characteristics** | | | **Intervention effect on depression** |
| --- | --- | --- | --- | --- | --- | --- | --- | --- | --- |
| **Study & Location** | **Focus on depression (secondary or unspecified)** | **Sample size** | **Study design** | **Age  (Mean ±SD**  **or %)** | **Depression measure** | **Primary intervention outcome(s) (excludes depression)** | **Intervention**  **description/ type** | **Intervention duration** | **Significant positive (i.e. lower depression)/ Not significant^a^** |
| [Al Hayek et al. (1)](#_ENREF_1)/ Saudi Arabia | Unspecified | 104 | Prospective intervention study | 57.3 ±14.4 | HADS | Metabolic control (diabetes self-management, medication adherence), psychological well‑being | Diabetes education programs (delivered by nurse-trained diabetes health educations + one‑on‑one counseling sessions with a doctor) | 12 sessions/  6 months | Significant positive/ 6 months |
| [Allen et al. (2)](#_ENREF_2)/ USA | Unspecified | 29/  CGM + Problem solving: 14  CGM + Education: 15 | Pre-test post-test randomized design | All: 53.0  ICGM + Problem solving): 52.2 ±6.5 | CES-D | Feasibility and acceptability, PA, diet, problem-solving skills, self-efficacy for PA, HbA1c, weight, blood pressure | CGM and problem solving counseling intervention for PA | 90 minute counselling session at week 4 | Not significant |
| [Araki et al. (3)](#_ENREF_3)/ Japan | Unspecified | 1173/  Interv: 585 | RCT | 72.0 | GDS-15 (DGDS ≥ 5) | Glycemic control, dyslipidemia, hypertension, obesity, diabetic complications and atherosclerotic disease | Long-term, multiple risk factor intervention (HbA1c levels, blood pressure, triglycerides) | Unclear | Unclear |
| [Aylin et al. (4)](#_ENREF_4)/ Turkey | Unspecified | 38/  Interv: 18 | Prospective intervention study | 54.3 ±8 | CES-D | Glycemic and metabolic control (i.e., HbA1c, cholesterol, triglycerides, etc.), quality of life | Combined resistance and home-based walking exercise | 8 weeks | Not significant |
| [Berger et al. (5)](#_ENREF_5)/ Israel | Unspecified | 11 | Pilot study (pre-test post-test design) | Range: 55-73 | BDI | Biomedical measures (HbA1c and blood lipids),  psychological measures (e.g., anxiety, hostility) | Integrative interdisciplinary group intervention program (nutritional instruction and stress-reduction techniques such as cognitive-behavioral skills, relaxation, and exercise) | 10 weeks | Significant positive |
| [Bradley et al. (6)](#_ENREF_6)/ USA | Unspecified | 40/  (329 in usual care control) | One year observational cohort study with intervention | Interv: 57.6 ±7.6, Control: 54.3 ±8.2 | PHQ-8 (threshold ≥ 10) | Patient-reported outcomes (PROs) (i.e., frequency of self-care activities, change) and clinical outcomes (i.e., change in HbA1c) | Up to 8 visits of adjunctive naturopathic care (ANC) for primary care patients with inadequately controlled T2D | 12 months | Significant positive |
| [Castillo et al. (7)](#_ENREF_7)/ USA | Secondary | 70 | Pilot study (pretest-posttest) | 58.2 ±13.1 | PHQ-9 | Glycemic control and self-management behaviors, clinical variables (various) | A linguistically and culturally appropriate diabetes group education program delivered by community health workers in community settings (Hispanics/Latinos) | 10 weeks/  2-hour sessions | Positive trend |
| [Chai et al. (8)](#_ENREF_8)/ China | Unspecified | 118 | Randomized intervention study | Interv: 55.0 ±7.0 Control: 53.0 ±9.0 | Self-rating Depression Scale (SDS) (Zung) | Anxiety, fasting blood glucose, HbA1c | Self-management education using Problem Based Learning (PBL) | 6 months | Significant positive/ 6 months |
| [Chen et al. (9)](#_ENREF_9)/ China | Unspecified | 1200 | Prospective intervention study | 50.5 ±14.2 | CES-D | Diabetes-specific emotional distress | Standard diabetes education program in newly diagnosed diabetics | 2 weeks | Significant positive**/**  Differences based on severity of symptoms |
| [Chew et al. (10)](#_ENREF_10)/ Malaysia | Secondary | 124 | Cluster RCT | Interv: 27-35 (median=35) Control: 24-44 (median=32) | PHQ-9 | Diabetes distress | Structured, value-based, emotion-focused educational program (VEMOFIT), delivered in primary care | 4 sessions/6 weeks with booster sessions 3 months later | Not significant |
| [Delevatti et al. (11)](#_ENREF_11)/ Brazil | Secondary | 35 | Randomized clinical trial | Aquatic: 54.2 ±8.3  Dry-land: 59.2 ±6.9 | BDI | Quality of life | Two aerobic training models in water and on dry-land | 12 weeks | Not significant |
| [DuBois et al. (12)](#_ENREF_12)/ USA | Secondary | 12 | Pilot, feasibility study (proof of concept) | 61.4 ±7.0 | HADS | Feasibility, acceptability | Telephone-based Positive psychology intervention | 12 weeks | Positive (moderate effect size) |
| [Fiocco et al. (13)](#_ENREF_13)/ Canada | Unspecified | 17 | Pilot study (pre-test, post-test design) | 57 (Standard error=1.88) | CES-D | Cardiovascular and metabolic measures and cognitive function | Diabetes exercise and healthy lifestyle service; weekly sessions of 60-minute exercise (aerobic and resistance) and 30-minute lectures with an educational component) | 24 weeks | Significant positive |
| [Friis et al. (14)](#_ENREF_14)/ New Zealand | Unspecified | 63 | RCT | All: 44.37 ±15.62 Interv: 42.16 ±17.70 Control: 46.65 ±16.44 | PHQ-9 | Self-compassion, diabetes distress, HbA1c | Mindful self-compassion: a protocol-standardized intervention aimed at increasing mindfulness and self-compassion and reducing the suffering associated with experiential avoidance | 8 weeks | Significant positive/ 3 months |
| [Gabbay et al. (15)](#_ENREF_15)/ USA | Unspecified | 545/  Interv: 232 | RCT (pragmatic) | Interv: 58.0 ±11.4  Control: 58.0 ±11.3 | CES-D (≥16) | HbA1c, LDL cholesterol, blood pressure, emotional distress, treatment satisfaction, self-care activities, diabetes-dependent quality of life | Addition of nurse case managers (coordinate health care needs) trained in Motivational Interviewing to usual care | 12 months + | Positive |
| [Glasgow et al. (16)](#_ENREF_16)/ USA | Secondary | 335/  Interv: 174 | Patient-level randomized practical clinical trial design | All: 61.5 ±11.3  Interv: 62.0 ±11.7  Control: 61.0 ±11.0 | PHQ-9 | Dietary behaviours (fat and fruit/vegetable intake), HbA1c, lipids, weight, quality of life | Social cognitive theory-based tailored self-management (TSM) delivered via computer program and health coaching sessions | 2 months | Positive trend |
| [Glasgow et al. (17)](#_ENREF_17)/ USA | Unspecified | 320/  Interv: NA | RCT | 59 ±9.2 | CES-D | Behavioral (e.g., dietary), biological (e.g., HbA1c), and psychosocial outcomes (e.g., depression) | “Diabetes Network (D-Net)” Internet-based tailored self-management (guided by self-efficacy theory);  Peer support intervention guided by social support theory | Unclear/  Evaluation at 10 months | Significant positive |
| [Holland-Carter et al. (18)](#_ENREF_18)/ USA | Secondary | 563 | Multi-site RCT | All: 55.1 ±9.1  Interv: 55.2 ±8.9  Standard care: 54.9 ±9.3 | PHQ-9 | HbA1c, weight change | Commercially available weight loss program (Weight Watchers; WW), enhanced with diabetes-specific counseling | 12 months | Not significant |
| [Hung et al. (19)](#_ENREF_19)/ Taiwan | Secondary | 95 | Quasi-experimental pre-post design | All: 61.3 ±8.0  Interv: 63.9 ±9.3 Control: 58.5 ±9.1 | Taiwanese Depression Questionnaire (TDQ) | Weekly SMBG times and biochemical parameters (BMI, HbA1c, fasting plasma glucose) | Diabetes Conversation Map Program (DCMP): patient-centered, conversation-based health education program | 7 weeks | Not significant |
| [Inouye et al. (20)](#_ENREF_20)/ USA | Unspecified | 207/  Interv: 104 | Randomized controlled clinical trial with double blind | All: 57.3 ±10.9  Interv: 57.0 ±11.1  Control: 57.8 ±10.8 | CES-D | Quality of life, general health perceptions, diabetes self-efficacy, glycemic control metabolic parameters. | Cognitive behavioral therapy (6 sessions) (i.e., stress management, biofeedback, assisted relaxation, mood management, cognitive restructuring, etc.) | 6 weeks | Significant positive/ Not maintained (12 months) |
| [Karlsen et al. (21)](#_ENREF_21)/  Norway | Unspecified | 63/  Interv: 31 | RCT | Interv: 49.2 ±14.7  Control: 48.6 ±10.3 | Short Zung Symptoms of Depression Rating Scale/ 10 questions | Stress, coping, psychological well-being, metabolic control | A group counselling programme based on Cognitive Behavior Theory | 12 months/  9 sessions (1.5 hours) | Not significant |
| [Kempf and Martin (22)](#_ENREF_22)/ Germany | Secondary | 220/  Interv: 120 | RCT | Interv: 62 ±11  Control: 60 ±9 | CES-D (German version), PAID, and WHO-5 | HbA1c | Autonomous use of the  interactive exercise game Wii Fit Plus | 12 weeks | Not significant/  Significant positive for WHO-5 only |
| [Kempf et al. (23)](#_ENREF_23)/ Germany | Unspecified | 228 (2-year FU)/  (Interv at baseline: 327) | Intervention study (pre-post with FU) | 59.8 ±8.6 | CES-D (score > 23) | Glucometabolic control (i.e., HbA1c), quality of life | Self-monitoring of blood glucose (SMBG) lifestyle intervention | 12 weeks/  2 year FU | Not significant |
| [Khunti et al. (24)](#_ENREF_24)/ UK | Secondary | 731/  Interv: 332 | RCT | All: 60.1 ±11.8, Interv: 59.4 ±11.6  Control: 61.01 ±12.1 | HADS | HbA1c | A single structured group education and self-management program delivered in the community by two trained healthcare professional educators | 6 hours /  2 half days or one full day | Not significant |
| [Li et al. (25)](#_ENREF_25)/ Canada | Secondary | 140 | RCT | All: 57.43 ±11.01 Interv: 56.69 ±11.16  Control: 58.23 ±10.87 | PHQ-9 | Glycated hemoglobin (A1C) diabetes distress | Nurse case management (NCM) intervention with 3 core components: 1) self-management and education, 2) monitoring and algorithm-driven treatment adjustment, 3) care coordination | 6 months | Significant positive |
| [Lin et al. (26)](#_ENREF_26)/ Taiwan | Unspecified | 13 | Prospective intervention study | 48.2 ±4.1 | BDI | Insulin sensitivity, quality of life | Aerobic exercise training | 12 weeks | Significant positive |
| [Lincoln et al. (27)](#_ENREF_27)/ USA | Primary (with quality of life) | 58/  Interv: 29 | RCT | Interv: 66.0 ±7.9 Control: 66.6 ±7.4 | GDS | Glycemic/metabolic control, mental component summary score of SF-36 | High-intensity progressive resistance exercise training | 16 weeks/  3 times/week | Significant positive |
| [Lorig et al. (28)](#_ENREF_28)/ USA | Unspecified | 352/  Interv: 186 | RCT | Interv: 67.7 ±11.9  Control: 65.4 ±11.4 | PHQ-9 | Health status, health/self-management behaviors, health care utilization, self-efficacy | Community-based & interactive peer-led diabetes self-management program (DSMP) | 6 weeks/  2.5 hours weekly | Significant positive |
| [Ma et al. (29)](#_ENREF_29)/ USA | Unspecified | 13 | Pilot study (pre-test post-test design) | 57.9 ±10.4 | CES-D | Feasibility, HbA1c, glycemic index and load, dietary intake, body weight, quality of life | Nutritionist-delivered low-glycemic index (GI) dietary intervention | 6 months | Not significant |
| [Malanda et al. (30)](#_ENREF_30)/ Netherlands | Secondary | 181/  Blood monitoring: 60,  Urine monitoring: 59 | Three-armed RCT | 61.8 ±7.6 | PHQ-9 | Diabetes-specific distress and self-efficacy, HbA1c, treatment satisfaction | Self-monitoring of glucose in blood or urine after individual training (“IN CONTROL” trial) | 12 months | Not significant |
| [McKay et al. (31)](#_ENREF_31)/USA, Canada | Unspecified | 68/  Interv: 35 | Randomized controlledpilot study | 52.3 | CES-D | Physical activity | Diabetes Network (D-Net) Active Lives physical activity  Intervention/ Internet-delivered Intervention | 8 weeks | Not significant |
| [Mons et al. (32)](#_ENREF_32)/ Germany | Secondary | 204/  Interv: 103 | RCT | Interv: 68.0 ±17  Control: 67.0 ±15 | GDS (15 items) | HbA1c | Supportive telephone-based counseling sessions led by practice nurses | 12 months | Significant positive/ Not maintained (18 months) |
| [Packer et al. (33)](#_ENREF_33)/ Australia | Unspecified | 458/  Living with Diabetes: 222 Living life with chronic condition: 236 | Quasi-experimental design | Diabetes: 60.0 ±11.0  Chronic: 70.1 ±10.5 | PHQ-9 | Self-management knowledge and Skills, health related quality of life, social isolation, loneliness; self-efficacy, health  behaviours | “Living with Diabetes”: interactive, behaviourally based group intervention toimprove self management and facilitate lifestyle modification (structured protocol and manual) | 6 weeks | Significant positive (both groups)/  Not maintained for Living with Diabetes |
| [Pauley et al. (34)](#_ENREF_34)/  Canada | Secondary | 94 | RCT | Interv: 65.1 ±13.2 Control: 66.9 ±11.7 | HADS | Diabetes Self-Efficacy and Insulin Management Diabetes Self-Efficacy | Paraprofessional-led diabetes self-management coaching | 6 weeks | Not significant |
| [Quinn et al. (35)](#_ENREF_35)/ USA | Secondary | 163/  Coach-only: 23, Coach PCP portal: 22, Coach PCP portal & decision support: 62 | Cluster-randomized clinical trial | 52.8  Range: 52.0-53.7 | PHQ-9 | HbA1c | The Mobile Diabetes Intervention Study: diabetes coaching system (and provider clinical decision support), using mobile phones and patient/ provider portals for patient-specific treatment and communication | 12 months | Not significant |
| [Quinn et al. (36)](#_ENREF_36)/ USA | Unspecified | 7 | Pilot study (prospective) | 70.3 ±3.2 | PHQ-9 | Use data for Patient-Coaching System (PCS), self-efficacy,  energy/fatigue, cognitive status, diabetes symptoms, overall health | Mobile diabetes coaching intervention for patients over 65years of age (patient-coaching system–version 2 [PCS]): communication software using patient self-care data and automated messages | 4 weeks | Significant positive |
| [Rosal et al. (37)](#_ENREF_37)/ USA | Unspecified | 25/  Interv: 15 | RCT | All: 62.6 ±8.6  Interv: 62.7 ±8.1  Control: 62.4 ±9.7 | CES-D | Physiological (e.g., HbA1c), Behavioral (e.g., dietary recall, PA), psychosocial (i.e., diabetes knowledge, quality of life) | Self-management intervention (cognitive behavioural theory based) targeting knowledge, attitudes, and skills; culturally specific and literacy sensitive | ~ 10 weeks/  10 weekly sessions (2.5-3 hours) | Significant positive |
| [Rosal et al. (38)](#_ENREF_38)/ USA | Unspecified | 89/  Face to face: 43,  Virtual reality: 46 | Randomized clinical trial | Total: 52 ±10  Face to face : 52 ±11  Virtual world: 53 ±10 | CES-D | Clinical measures (i.e., HbA1c, blood pressure, height, weight, waist circumference), self-management behaviors (diet, PA, blood glucose self-monitoring, medication adherence), social support, perceived stress, quality of life | Virtual reality and group-based diabetes self-management program adapted from Power to Prevent, a behavior-change in-person group program for African Americans with diabetes or pre-diabetes | 8 weekly sessions | Positive trend/  Face to face marginally superior to virtual word |
| [Rosenzweig et al. (39)](#_ENREF_39)/ USA | Unspecified | 11 | Prospective observational study with intervention | 59.2 ±2.57 | Symptom Checklist 90-Revised | HbA1c, blood pressure, body weight, anxiety, somatization, general psychological distress | Mindfulness-Based Stress Reduction (MBSR) | 8 weeks/  Weekly 150-minute sessions + 7-hour weekend session | Significant positive |
| [Rubin et al. (40)](#_ENREF_40)/ USA | Secondary | 5145/  Interv: 2570 | RCT (multisite) | 58.37 ±6.8 | BDI-1A (score ≥10) | Weight loss, composite of death from cardiovascular causes, nonfatal myocardial infarction, nonfatal stroke, or hospitalization for angina | Look AHEAD (Action for Health in Diabetes)  trial : long-term intensive  lifestyle intervention  to achieve weight loss | 9 years/  1-Weekly sessions (6 months); 2-3x/ month (6 months); 3-Monthly/weekly sessions offered | Significant positive/  Differences based on severity of symptoms |
| [Sacco et al. (41)](#_ENREF_41)/ USA | Unspecified | 62/  Interv: 31 | RCT | 52 ±8.6 | PHQ-9 | Diabetes adherence, glycemic control, diabetes-related medical symptoms | Brief, regular, proactive, telephone ‘‘coaching’’ intervention delivered by paraprofessionals | 6 months | Significant positive |
| [Satish and Lakshmi (42)](#_ENREF_42)/ India | Unspecified | 90 | Prospective intervention study | 54.59 ±10.15 | BDI | Self-management approach to achieve glycemic control and psychological wellbeing | An individualized approach of providing yoga support | 12 sessions | Not significant |
| [Spencer et al. (43)](#_ENREF_43)/USA | Secondary | 222 | Randomized intervention design | 48.9 ±10.6 | PHQ-9 | HbA1c | Community health worker (CHW) diabetes self-management education (DSME) program with additional peer-led (PL) maintenance intervention | 6 months/ PL additional 6-18 months | Significant positive |
| [Steinhardt et al. (44)](#_ENREF_44)/ USA | Unspecified | 65/  Interv: 32 | Quasi-experimental design | Interv:  6.3% = 30-40  53.1% = 45-59  34.4% = 60-74  6.3% = 75+) | CES-D | Feasibility, diabetes knowledge, psychological well-being (resilience, quality of life, perceived stress), diabetes self-management (e.g., PA), body fat, BMI, waist circumference, HbA1C, CVD indicators (e.g., blood pressure, LDL & HDL cholesterol) | Resilience-based diabetes self-management education (RB-DSME) program | 8 weeks/  8 weekly classes | Not significant |
| [Swoboda et al. (45)](#_ENREF_45)/ USA | Unspecified | 54 | Randomized pretest-posttest control group design | Interv: 56.76 ±7.35  Control: 55.41 ±7.82 | PHQ-8 | Diet quality, diabetes self-efficacy and diabetes distress | Decision support and goal-setting telephone coaching intervention | 16 weeks | Significant positive |
| [Toobert et al. (46)](#_ENREF_46)/ USA | Secondary | 279/  Interv: 163 | Randomized intervention study | Interv: 61.1 ±8.0  Control: 60.7 ±7.8 | CES-D | Behavioral (dietary, PA, stress management, social desirability); psychosocial (social resources, problem-solving, self-efficacy, perceived stress, quality of life) | Mediterranean Lifestyle Program (MLP): Theory-based comprehensive lifestyle management to reduce coronary heart disease risk in postmenopausal women with T2D | 6 months/  (+ 18-month maintenance intervention) | Not significant |
| [Trief et al. (47)](#_ENREF_47)/ USA | Secondary | 268 | Three-armed randomized controlled trial | 56.8 ±10.9 | PHQ-8 | HbA1c | Telephonic couples behavioral intervention | 12 months | Significant positive |
| [van der Wulp et al. (48)](#_ENREF_48)/ Netherlands | Secondary | 119/  Interv: 59 | RCT | (Median)  All: 61.0  Interv: 60.0  Control: 62.5 | CES-D | Self-efficacy | Peer led self-management coaching programme (for recently diagnosed T2D patients) | 3 monthly home visits | Not significant/  negative |
| [Wang et al. (49)](#_ENREF_49)/ USA | Secondary | 252/  Interv: 124 | Randomized intervention study | 16.3% = 18-44, 29.8% = 45-54, 32.9% = 55-64, 21% = 65 +) | CES-D | Behavioral (dietary quality and intake, PA), and clinical (HbA1c) outcomes | “Latinos en Control”: Culturally and literacy-tailored group-based self-management intervention targeting knowledge, self-efficacy, behavior | 12 months/  (12 weekly sessions + 8 monthly sessions) | Significant positive |
| [Wayne et al. (50)](#_ENREF_50)/ Canada | Secondary | 97/  Interv: 48 | Noninferiority pragmatic RCT with a 1:1 allocation | All: 53.2 ±11.3  Interv: 53.1 ±10.9  Control: 53.3 ±11.9 | HADS | HbA1c | Mobile phone-based health coaching intervention (behaviour change protocol) | 6 months | Not significant/ Positive for interv and control groups |
| [Welch et al. (51)](#_ENREF_51)/ USA | Unspecified | 39/  Interv: 21 | RCT | Interv: 54.4 ±10.4  Control: 57.5 ±9.5 | PHQ-9/  Cut-point = 10 | Clinical data (adherence to clinical practice guidelines), levels of diabetes distress, depression, and treatment satisfaction | Bicultural/ bilingual nurse-led diabetes care program (Comprehensive Diabetes Management Program, [CDMP]): interactive, web-based tool based on American Diabetes Association practice guidelines | 12 months/  7 visits | Not significant |
| [Welschen et al. (52)](#_ENREF_52)/ Netherlands | Unspecified | 154/  Interv: 76 | RCT | Interv: 60.5 ±9.4  Control: 61.2 ±8.8 | CES-D | Coronary heart disease risk, clinical characteristics, lifestyle (PA, eating behaviour, smoking), quality of life | Cognitive behavioural treatment (CBT) aimed at changing lifestyle in the context of managed care | 3-6 sessions of 30 minutes | Significant positive/  Not maintained (6 months) |
| [Williams et al. (53)](#_ENREF_53)/ Secondary | Secondary | 886/  Interv: 469 | RCT | Interv: 62 ±1.4  Control: 64 ±1.3 | PHQ-9 (>10) | Number of recommended laboratory screenings and recommendedpatient-centered care activities (see [Glasgow et al. (54)](#_ENREF_54)) | A computer-assisted, patient-centered intervention consistent with the Chronic Care Model; self-management action planning related to dietary, PA and/or smoking with tailored feedback | 12 months/  2 visits | Positive/  Group differences not significant |
| [Wu et al. (55)](#_ENREF_55)/ Taiwan | Unspecified | 145/  Interv: 72 | Quasi-experimental design | Interv: 64.8 ±9.8  Control: 64.1 ±10.1 | CES-D | Health-related quality of life, social support, well-being | Self-management programme (Self-Efficacy Enhancing Intervention Programme [SEEIP], including booklet, DVD and self-efficacy sessions | 4 weeks/  Weekly session | Not significant |
| [Wu et al. (56)](#_ENREF_56)/ Taiwan | Unspecified | 228/  Interv: 147 | Quasi-experimental design | 60.83 ±12.43 | Depression, Anxiety and Stress Scale (DASS) | Physiological indicators (BMI, Waistline circumference, HbA1C, anxiety, self-efficacy, self-care, well-being | Diabetes self-management programme administered by healthcare workers who have received a special leader training programme | 4 weeks/  weekly course | Significant positive |
| [Yucel and Uysal (57)](#_ENREF_57)/ Turkey | Unspecified | 45/  Interv: 24 | Prospective and randomized study | Interv: 58.50 ±7.00  Control: 53.50 ±9.00 | HADS | Glycemic control, anxiety, quality of life | Pilates­based mat exercise (PBME) | 12 weeks/  3 weekly sessions (~ 1 hour) | Significant positive |

Interv = Intervention; PCP = Primary Care Providers; CGM = Continuous Glucose Monitoring; RCT = Randomized controlled trial; FU = Follow-up; CES-D = Center for Epidemiology Studies Short Depression Scale; PHQ-9 = Patient Health Questionnare-9; GDS = Geriatric Depression Scale; HADS = Hospital anxiety depression scale; BDI = Beck Depression Inventory; PAID = Problem Areas in Diabetes Scale; WHO-5 = World Health Organization Well-Being Questionnaire; BMI = Body Mass Index; CVD = Cardiovascular Disease; PA = Physical Activity; HbA1c = haemoglobin A1c or glycated haemoglobin; SF-36 = 36-Item Short Form Health Survey; LDL/HDL = Low/High Density Lipoprotein; T2D = Type 2 Diabetes; SMBG = Self-Monitoring of Blood Glucose

^a^A significant positive effect corresponds to a reduction in depression levels (symptoms, scores) in intervention compared to control or alternative group in multi-group studies and pre-post changes in single-group studies.

References:

1. Al Hayek AA, Robert AA, Al Dawish MA, Zamzami MM, Sam AE, Alzaid AA. Impact of an education program on patient anxiety, depression, glycemic control, and adherence to self-care and medication in Type 2 diabetes. *Journal of family & community medicine* (2013) 20(2):77-82. doi: 10.4103/2230-8229.114766. PubMed PMID: 23983558; PubMed Central PMCID: PMC3748651.

2. Allen N, Whittemore R, Melkus G. A continuous glucose monitoring and problem-solving intervention to change physical activity behavior in women with type 2 diabetes: a pilot study. *Diabetes technology & therapeutics* (2011) 13(11):1091-9. doi: 10.1089/dia.2011.0088. PubMed PMID: 21919735.

3. Araki A, Iimuro S, Sakurai T, Umegaki H, Iijima K, Nakano H, et al. Long-term multiple risk factor interventions in Japanese elderly diabetic patients: the Japanese Elderly Diabetes Intervention Trial--study design, baseline characteristics and effects of intervention. *Geriatrics & gerontology international* (2012) 12 Suppl 1:7-17. doi: 10.1111/j.1447-0594.2011.00808.x. PubMed PMID: 22435936.

4. Aylin K, Arzu D, Sabri S, Handan TE, Ridvan A. The effect of combined resistance and home-based walking exercise in type 2 diabetes patients. *International journal of diabetes in developing countries* (2009) 29(4):159-65. doi: 10.4103/0973-3930.57347. PubMed PMID: 20336198; PubMed Central PMCID: PMC2839130.

5. Berger R, Gidron Y, Harman-Boehm I, Dekel G, Shwartzman P, Sarid O. An Integrative Cognitive- Behavioral Approach for the Treatment for Type 2 Diabetes Miletus: A Pilot Study. *The Endocrinologist* (2007) 17(2):122-6. doi: 10.1097/01.ten.0000261461.61325.90.

6. Bradley R, Sherman KJ, Catz S, Calabrese C, Oberg EB, Jordan L, et al. Adjunctive naturopathic care for type 2 diabetes: patient-reported and clinical outcomes after one year. *BMC complementary and alternative medicine* (2012) 12:44. doi: 10.1186/1472-6882-12-44. PubMed PMID: 22512949; PubMed Central PMCID: PMC3403984.

7. Castillo A, Giachello A, Bates R, Concha J, Ramirez V, Sanchez C, et al. Community-based Diabetes Education for Latinos: The Diabetes Empowerment Education Program. *The Diabetes educator* (2010) 36(4):586-94. doi: 10.1177/0145721710371524. PubMed PMID: 20538970.

8. Chai S, Yao B, Xu L, Wang D, Sun J, Yuan N, et al. The effect of diabetes self-management education on psychological status and blood glucose in newly diagnosed patients with diabetes type 2. *Patient education and counseling* (2018) 101(8):1427-32. doi: 10.1016/j.pec.2018.03.020. PubMed PMID: 29622281.

9. Chen B, Zhang X, Xu X, Lv X, Yao L, Huang X, et al. Diabetes education improves depressive state in newly diagnosed patients with type 2 diabetes. *Pakistan journal of medical sciences* (2013) 29(5):1147-52. PubMed PMID: 24353709; PubMed Central PMCID: PMC3858924.

10. Chew BH, Vos RC, Stellato RK, Ismail M, Rutten G. The effectiveness of an emotion-focused educational programme in reducing diabetes distress in adults with Type 2 diabetes mellitus (VEMOFIT): a cluster randomized controlled trial. *Diabetic Medicine* (2018) 35(6):750-9. doi: 10.1111/dme.13615. PubMed PMID: 29505098.

11. Delevatti RS, Schuch FB, Kanitz AC, Alberton CL, Marson EC, Lisboa SC, et al. Quality of life and sleep quality are similarly improved after aquatic or dry-land aerobic training in patients with type 2 diabetes: A randomized clinical trial. *Journal of science and medicine in sport* (2018) 21(5):483-8. doi: 10.1016/j.jsams.2017.08.024. PubMed PMID: 28935128.

12. DuBois CM, Millstein RA, Celano CM, Wexler DJ, Huffman JC. Feasibility and Acceptability of a Positive Psychological Intervention for Patients With Type 2 Diabetes. *The primary care companion for CNS disorders* (2016) 18(3). doi: 10.4088/PCC.15m01902. PubMed PMID: 27733954; PubMed Central PMCID: PMC5035810.

13. Fiocco AJ, Scarcello S, Marzolini S, Chan A, Oh P, Proulx G, et al. The effects of an exercise and lifestyle intervention program on cardiovascular, metabolic factors and cognitive performance in middle-aged adults with type II diabetes: a pilot study. *Canadian journal of diabetes* (2013) 37(4):214-9. doi: 10.1016/j.jcjd.2013.03.369. PubMed PMID: 24070883.

14. Friis AM, Johnson MH, Cutfield RG, Consedine NS. Kindness Matters: A Randomized Controlled Trial of a Mindful Self-Compassion Intervention Improves Depression, Distress, and HbA1c Among Patients With Diabetes. *Diabetes care* (2016) 39(11):1963-71. doi: 10.2337/dc16-0416. PubMed PMID: 27335319.

15. Gabbay RA, Anel-Tiangco RM, Dellasega C, Mauger DT, Adelman A, Van Horn DH. Diabetes nurse case management and motivational interviewing for change (DYNAMIC): results of a 2-year randomized controlled pragmatic trial. *Journal of diabetes* (2013) 5(3):349-57. doi: 10.1111/1753-0407.12030. PubMed PMID: 23368423; PubMed Central PMCID: PMC3679203.

16. Glasgow RE, Nutting PA, Toobert DJ, King DK, Strycker LA, Jex M, et al. Effects of a brief computer-assisted diabetes self-management intervention on dietary, biological and quality-of-life outcomes. *Chronic illness* (2006) 2(1):27-38. doi: 10.1177/17423953060020011001. PubMed PMID: 17175680.

17. Glasgow RE, Boles SM, McKay HG, Feil EG, Barrera M, Jr. The D-Net diabetes self-management program: long-term implementation, outcomes, and generalization results. *Preventive medicine* (2003) 36(4):410-9. PubMed PMID: 12649049.

18. Holland-Carter L, Tuerk PW, Wadden TA, Fujioka KN, Becker LE, Miller-Kovach K, et al. Impact on psychosocial outcomes of a nationally available weight management program tailored for individuals with type 2 diabetes: Results of a randomized controlled trial. *Journal of diabetes and its complications* (2017) 31(5):891-7. doi: 10.1016/j.jdiacomp.2017.01.022. PubMed PMID: 28319001.

19. Hung JY, Chen PF, Livneh H, Chen YY, Guo HR, Tsai TY. Long-term effectiveness of the Diabetes Conversation Map Program: A prepost education intervention study among type 2 diabetic patients in Taiwan. *Medicine (Baltimore)* (2017) 96(36):e7912. doi: 10.1097/MD.0000000000007912. PubMed PMID: 28885345.

20. Inouye J, Li D, Davis J, Arakaki R. Psychosocial and Clinical Outcomes of a Cognitive Behavioral Therapy for Asians and Pacific Islanders with Type 2 Diabetes: A Randomized Clinical Trial. *Hawai'i Journal of Medicine & Public Health* (2015) 74(11):360-8. PubMed PMID: 26568899; PubMed Central PMCID: PMC4642496.

21. Karlsen B, Idsoe T, Dirdal I, Rokne Hanestad B, Bru E. Effects of a group-based counselling programme on diabetes-related stress, coping, psychological well-being and metabolic control in adults with type 1 or type 2 diabetes. *Patient education and counseling* (2004) 53(3):299-308. doi: 10.1016/j.pec.2003.10.008. PubMed PMID: 15186867.

22. Kempf K, Martin S. Autonomous exercise game use improves metabolic control and quality of life in type 2 diabetes patients - a randomized controlled trial. *BMC endocrine disorders* (2013) 13:57. doi: 10.1186/1472-6823-13-57. PubMed PMID: 24321337; PubMed Central PMCID: PMC3880220.

23. Kempf K, Kruse J, Martin S. ROSSO-in-praxi follow-up: long-term effects of self-monitoring of blood glucose on weight, hemoglobin A1c, and quality of life in patients with type 2 diabetes mellitus. *Diabetes technology & therapeutics* (2012) 14(1):59-64. doi: 10.1089/dia.2011.0116. PubMed PMID: 21988274.

24. Khunti K, Gray LJ, Skinner T, Carey ME, Realf K, Dallosso H, et al. Effectiveness of a diabetes education and self management programme (DESMOND) for people with newly diagnosed type 2 diabetes mellitus: three year follow-up of a cluster randomised controlled trial in primary care. *Bmj* (2012) 344:e2333. doi: 10.1136/bmj.e2333. PubMed PMID: 22539172; PubMed Central PMCID: PMC3339877.

25. Li D, Elliott T, Klein G, Ur E, Tang TS. Diabetes Nurse Case Management in a Canadian Tertiary Care Setting: Results of a Randomized Controlled Trial. *Canadian journal of diabetes* (2017) 41(3):297-304. doi: 10.1016/j.jcjd.2016.10.012. PubMed PMID: 28318938.

26. Lin C-H, Ho C-W, Chen L-C, Chang C-C, Wang Y-W, Chiou C-P, et al. Effects of a 12-week exercise training on insulin sensitivity, quality of life, and depression status in patients with type 2 diabetes. *Journal of Medical Sciences* (2017) 37(6):227-36. doi: 10.4103/jmedsci.jmedsci_68_17.

27. Lincoln AK, Shepherd A, Johnson PL, Castaneda-Sceppa C. The impact of resistance exercise training on the mental health of older Puerto Rican adults with type 2 diabetes. *The journals of gerontology Series B, Psychological sciences and social sciences* (2011) 66(5):567-70. doi: 10.1093/geronb/gbr034. PubMed PMID: 21571703; PubMed Central PMCID: PMC3155029.

28. Lorig K, Ritter PL, Villa FJ, Armas J. Community-based peer-led diabetes self-management: a randomized trial. *The Diabetes educator* (2009) 35(4):641-51. doi: 10.1177/0145721709335006. PubMed PMID: 19407333.

29. Ma Y, Olendzki BC, Chiriboga D, Rosal M, Sinagra E, Crawford S, et al. PDA-assisted low glycemic index dietary intervention for type II diabetes: a pilot study. *European journal of clinical nutrition* (2006) 60(10):1235-43. doi: 10.1038/sj.ejcn.1602443. PubMed PMID: 16708066.

30. Malanda UL, Bot SD, Kostense PJ, Snoek FJ, Dekker JM, Nijpels G. Effects of self-monitoring of glucose on distress and self-efficacy in people with non-insulin-treated Type 2 diabetes: a randomized controlled trial. *Diabetic Medicine* (2016) 33(4):537-46. doi: 10.1111/dme.12849. PubMed PMID: 26171942.

31. McKay HG, King D, Eakin EG, Seeley JR, Glasgow RE. The diabetes network internet-based physical activity intervention: a randomized pilot study. *Diabetes care* (2001) 24(8):1328-34. PubMed PMID: 11473065.

32. Mons U, Raum E, Kramer HU, Ruter G, Rothenbacher D, Rosemann T, et al. Effectiveness of a supportive telephone counseling intervention in type 2 diabetes patients: randomized controlled study. *PloS one* (2013) 8(10):e77954. doi: 10.1371/journal.pone.0077954. PubMed PMID: 24205043; PubMed Central PMCID: PMC3813502.

33. Packer TL, Boldy D, Ghahari S, Melling L, Parsons R, Osborne RH. Self-management programs conducted within a practice setting: who participates, who benefits and what can be learned? *Patient education and counseling* (2012) 87(1):93-100. doi: 10.1016/j.pec.2011.09.007. PubMed PMID: 21992799.

34. Pauley T, Gargaro J, Chenard G, Cavanagh H, McKay SM. Home-based diabetes self-management coaching delivered by paraprofessionals: A randomized controlled trial. *Home health care services quarterly* (2016) 35(3-4):137-54. doi: 10.1080/01621424.2016.1264339. PubMed PMID: 27897469.

35. Quinn CC, Shardell MD, Terrin ML, Barr EA, Ballew SH, Gruber-Baldini AL. Cluster-randomized trial of a mobile phone personalized behavioral intervention for blood glucose control. *Diabetes care* (2011) 34(9):1934-42. doi: 10.2337/dc11-0366. PubMed PMID: 21788632; PubMed Central PMCID: PMC3161305.

36. Quinn CC, Khokhar B, Weed K, Barr E, Gruber-Baldini AL. Older Adult Self-Efficacy Study of Mobile Phone Diabetes Management. *Diabetes technology & therapeutics* (2015) 17(7):455-61. doi: 10.1089/dia.2014.0341. PubMed PMID: 25692373; PubMed Central PMCID: PMC4808269.

37. Rosal MC, Olendzki B, Reed GW, Gumieniak O, Scavron J, Ockene I. Diabetes self-management among low-income Spanish-speaking patients: a pilot study. *Annals of Behavioral Medicine* (2005) 29(3):225-35. doi: 10.1207/s15324796abm2903_9. PubMed PMID: 15946117.

38. Rosal MC, Heyden R, Mejilla R, Capelson R, Chalmers KA, Rizzo DePaoli M, et al. A Virtual World Versus Face-to-Face Intervention Format to Promote Diabetes Self-Management Among African American Women: A Pilot Randomized Clinical Trial. *JMIR research protocols* (2014) 3(4):e54. doi: 10.2196/resprot.3412. PubMed PMID: 25344620; PubMed Central PMCID: PMC4259910.

39. Rosenzweig S, Reibel DK, Greeson JM, Edman JS, Jasser SA, McMearty KD, et al. Mindfulness-based stress reduction is associated with improved glycemic control in type 2 diabetes mellitus: a pilot study. *Alternative therapies in health and medicine* (2007) 13(5):36-8. PubMed PMID: 17900040.

40. Rubin RR, Wadden TA, Bahnson JL, Blackburn GL, Brancati FL, Bray GA, et al. Impact of intensive lifestyle intervention on depression and health-related quality of life in type 2 diabetes: the Look AHEAD Trial. *Diabetes care* (2014) 37(6):1544-53. doi: 10.2337/dc13-1928. PubMed PMID: 24855155; PubMed Central PMCID: PMC4030096.

41. Sacco WP, Malone JI, Morrison AD, Friedman A, Wells K. Effect of a brief, regular telephone intervention by paraprofessionals for type 2 diabetes. *Journal of behavioral medicine* (2009) 32(4):349-59. doi: 10.1007/s10865-009-9209-4. PubMed PMID: 19365719.

42. Satish L, Lakshmi VS. Impact of individualized yoga therapy on perceived quality of life performance on cognitive tasks and depression among Type II diabetic patients. *International journal of yoga* (2016) 9(2):130-6. doi: 10.4103/0973-6131.183707. PubMed PMID: 27512320; PubMed Central PMCID: PMC4959323.

43. Spencer MS, Kieffer EC, Sinco B, Piatt G, Palmisano G, Hawkins J, et al. Outcomes at 18 Months From a Community Health Worker and Peer Leader Diabetes Self-Management Program for Latino Adults. *Diabetes care* (2018) 41(7):1414-22. doi: 10.2337/dc17-0978. PubMed PMID: 29703724; PubMed Central PMCID: PMC6014532.

44. Steinhardt MA, Brown SA, Dubois SK, Harrison L, Jr., Lehrer HM, Jaggars SS. A resilience intervention in African-American adults with type 2 diabetes. *American journal of health behavior* (2015) 39(4):507-18. doi: 10.5993/AJHB.39.4.7. PubMed PMID: 26018099.

45. Swoboda CM, Miller CK, Wills CE. Impact of a goal setting and decision support telephone coaching intervention on diet, psychosocial, and decision outcomes among people with type 2 diabetes. *Patient education and counseling* (2017) 100(7):1367-73. doi: 10.1016/j.pec.2017.02.007. PubMed PMID: 28215827.

46. Toobert DJ, Glasgow RE, Strycker LA, Barrera M, Jr., Ritzwoller DP, Weidner G. Long-term effects of the Mediterranean lifestyle program: a randomized clinical trial for postmenopausal women with type 2 diabetes. *The international journal of behavioral nutrition and physical activity* (2007) 4:1. doi: 10.1186/1479-5868-4-1. PubMed PMID: 17229325; PubMed Central PMCID: PMC1783667.

47. Trief PM, Fisher L, Sandberg J, Cibula DA, Dimmock J, Hessler DM, et al. Health and Psychosocial Outcomes of a Telephonic Couples Behavior Change Intervention in Patients With Poorly Controlled Type 2 Diabetes: A Randomized Clinical Trial. *Diabetes care* (2016) 39(12):2165-73. doi: 10.2337/dc16-0035. PubMed PMID: 27456837; PubMed Central PMCID: PMC5127234.

48. van der Wulp I, de Leeuw JR, Gorter KJ, Rutten GE. Effectiveness of peer-led self-management coaching for patients recently diagnosed with Type 2 diabetes mellitus in primary care: a randomized controlled trial. *Diabetic Medicine* (2012) 29(10):e390-7. doi: 10.1111/j.1464-5491.2012.03629.x. PubMed PMID: 22414198.

49. Wang ML, Lemon SC, Whited MC, Rosal MC. Who benefits from diabetes self-management interventions? The influence of depression in the Latinos en Control trial. *Annals of Behavioral Medicine* (2014) 48(2):256-64. doi: 10.1007/s12160-014-9606-y. PubMed PMID: 24664615.

50. Wayne N, Perez DF, Kaplan DM, Ritvo P. Health Coaching Reduces HbA1c in Type 2 Diabetic Patients From a Lower-Socioeconomic Status Community: A Randomized Controlled Trial. *Journal of medical Internet research* (2015) 17(10):e224. doi: 10.2196/jmir.4871. PubMed PMID: 26441467; PubMed Central PMCID: PMC4642794.

51. Welch G, Allen NA, Zagarins SE, Stamp KD, Bursell SE, Kedziora RJ. Comprehensive diabetes management program for poorly controlled Hispanic type 2 patients at a community health center. *The Diabetes educator* (2011) 37(5):680-8. doi: 10.1177/0145721711416257. PubMed PMID: 21918206.

52. Welschen LM, van Oppen P, Bot SD, Kostense PJ, Dekker JM, Nijpels G. Effects of a cognitive behavioural treatment in patients with type 2 diabetes when added to managed care; a randomised controlled trial. *Journal of behavioral medicine* (2013) 36(6):556-66. doi: 10.1007/s10865-012-9451-z. PubMed PMID: 23054175.

53. Williams GC, Lynch M, Glasgow RE. Computer-assisted intervention improves patient-centered diabetes care by increasing autonomy support. *Health Psychology* (2007) 26(6):728-34. doi: 10.1037/0278-6133.26.6.728. PubMed PMID: 18020845.

54. Glasgow RE, Nutting PA, King DK, Nelson CC, Cutter G, Gaglio B, et al. Randomized effectiveness trial of a computer-assisted intervention to improve diabetes care. *Diabetes care* (2005) 28(1):33-9. PubMed PMID: 15616230.

55. Wu SF, Liang SY, Wang TJ, Chen MH, Jian YM, Cheng KC. A self-management intervention to improve quality of life and psychosocial impact for people with type 2 diabetes. *Journal of clinical nursing* (2011) 20(17-18):2655-65. doi: 10.1111/j.1365-2702.2010.03694.x. PubMed PMID: 21605212.

56. Wu SF, Liang SY, Lee MC, Yu NC, Kao MJ. The efficacy of a self-management programme for people with diabetes, after a special training programme for healthcare workers in Taiwan: a quasi-experimental design. *Journal of clinical nursing* (2014) 23(17-18):2515-23. doi: 10.1111/jocn.12440. PubMed PMID: 24354787.

57. Yucel H, Uysal O. Pilates-Based Mat Exercises and Parameters of Quality of Life in Women With Type 2 Diabetes. *Iranian Red Crescent Medical Journal* (2016) 18(3):e21919. Epub 2016-03-02. doi: 10.5812/ircmj.21919.
