# Supplementary material for: Intervention Strategies for Prevention of Comorbid Depression Among Individuals With Type 2 Diabetes: A Scoping Review
Source: Front Public Health. 2019 Mar 5;7:35. doi: 10.3389/fpubh.2019.00035 (PMC6411710; doi:10.3389/fpubh.2019.00035)
Supplement: Supplementary file 2 [file Data_Sheet_1.docx]

Supplemental File 1

**Search strategy EMBASE & MEDLINE:**

1 *non insulin dependent diabetes mellitus/ (178982)

2 (Type* adj3 ("2" or "II" or two*) adj3 (diabete* or diabetic*)).tw. (252011)

3 (adult* onset* adj3 (diabete* or diabetic*)).tw. (1600)

4 ((Ketosis-resistant* or stable*) adj3 (diabete* or diabetic*)).tw. (1671)

5 ((Non-insulin* or Non insulin* or Noninsulin*) adj3 depend* adj3 (diabete* or diabetic*)).tw. (24838)

6 NIDDM.tw. (14647)

7 T2D.tw. (12977)

8 or/1-7 (312256)

9 *depression/ (179392)

10 *dysthymia/ (2179)

11 *mood disorder/ (14625)

12 (depress* or dysthym* or mood).ti. (289563)

13 (depression or depressive or depressed or dysthymic disorder* or mood disorder*).tw. (818631)

14 or/9-13 (871488)

15 8 and 14 (6509)

16 limit 15 to yr="2000 -Current" (6012)

17 *health education/ or *patient education/ or *patient counseling/ (130577)

18 *health promotion/ (71743)

19 *self care/ (27085)

20 exp *psychotherapy/ (225747)

21 *patient counseling/ or *counseling/ (33652)

22 *social support/ (36479)

23 intervention study/ (530345)

24 exp practice guideline/ (388081)

25 (psychoeducation or teach* or knowledge or inform).ti. (217574)

26 (program* or promot* or approach or strateg* or prevent* or intervention* or educat*).tw. (9454429)

27 (self-care or self-management or self-monitor* or self-help).tw. (76119)

28 ((health or patient or self) adj2 (knowledge or promotion or teach* or instruct* or learn* or information or coach*)).tw. (169309)

29 (treat* or manag* or recommendation* or guideline* or consensus or cpg* or pathway* or standard).tw. (14700369)

30 (cognitive or behavio?r or therapy or coach*).ti. (1598003)

31 (psychotherapy or psycho-therapy or cbt or counseling).tw. (202153)

32 ((cognitive behavio?ral or cognitive or behavio?r*) adj3 (therap* or treat*)).tw. (96496)

33 ((social or family or peer) adj3 support).tw. (83512)

34 ((depression or mood or depressive) adj3 (treat* or therapy)).tw. (69441)

35 or/17-34 (21472421)

36 16 and 35 (4468)

37 36 use emczd (2705) Lines 1-37 are the Embase Search

38 Diabetes Mellitus, Type 2/ (125258)

39 (Type* adj3 ("2" or "II" or two*) adj3 (diabete* or diabetic*)).tw. (252011)

40 (adult* onset* adj3 (diabete* or diabetic*)).tw. (1600)

Annotation: (adult onset* adj3 (diabete* or diabetic*)).tw.

41 ((Ketosis-resistant* or stable*) adj3 (diabete* or diabetic*)).tw. (1671)

42 ((Non-insulin* or Non insulin* or Noninsulin*) adj3 depend* adj3 (diabete* or diabetic*)).tw. (24838)

43 NIDDM.tw. (14647)

44 T2D.tw. (12977)

45 or/38-44 (315466)

46 Depression/ (376179)

47 Depressive Disorder/ (135336)

48 Mood Disorders/ (17681)

49 depressive disorder, major/ or dysthymic disorder/ (31281)

50 (depress* or dysthym* or mood).ti. (289563)

51 (depression* or depressive* or depressed or dysthymic disorder* or mood disorder*).tw. (827234)

52 or/46-51 (984227)

53 45 and 52 (7224)

54 limit 53 to yr="2000 -Current" (6677)

55 Patient Education as Topic/ (155831)

56 Health Education/ (142848)

57 Health Promotion/ (138875)

58 Self Care/ (66080)

59 exp Psychotherapy/ (386098)

60 Counseling/ (80835)

61 Social Support/ (124539)

62 Practice Guideline/ or Guideline/ (304517)

63 (psychoeducation* or teach* or knowledge or inform*).ti. (412431)

64 (program* or promot* or approach or strateg* or prevent* or intervention* or educat*).tw. (9454429)

65 (self-care or self-management or self-monitor* or self-help).tw. (76119)

Annotation: self-monitor*

66 ((health or patient or self) adj2 (knowledge or promotion or teach* or instruct* or learn* or information or coaching)).tw. (168911)

67 (treat* or manag* or recommendation* or guideline* or consensus or cpg* or pathway* or standard*).ti. (4183532)

68 (cognitive or behavio?r* or therapy or coach*).ti. (1759069)

69 (psychotherapy or psycho-therapy or cbt or counseling).tw. (202153)

70 ((cognitive behavio?ral or cognitive or behavio?r*) adj3 (therap* or treat*)).tw. (96496)

71 ((social or family or peer) adj3 support).tw. (83512)

72 ((depression or mood or depressive) adj3 (treat* or therapy)).tw. (69441)

73 or/55-72 (14787005)

74 54 and 73 (3980)

75 74 use prmz (1555) 38-75 Medline Search

76 37 or 75 (4260)

77 remove duplicates from 76 (3132) = number of citations after duplicates removed (Embase and Medline Combined)

**Search strategy PUBMED (without Medline)**

((((type II diabetes) OR type 2 diabetes) OR ((((diabet* AND type 2[Title/Abstract]) OR diabet* AND type II[Title/Abstract]) OR niddm[Title/Abstract]) OR t2d[Title/Abstract]))) AND ((((depression) OR depressive disorder*) OR dysthymic disorder) OR mood disorder*) AND (pubstatusaheadofprint OR publisher[sb] OR pubmednotmedline[sb])

**Search strategy Psychinfo**

--------------------------------------------------------------------------------

1 TYPE 2 DIABETES/ (2537)

2 (Type* adj3 ("2" or "II" or two*) adj3 (diabete* or diabetic*)).tw. (5768)

3 (adult* onset* adj3 (diabete* or diabetic*)).tw. (42)

4 ((Ketosis-resistant* or stable*) adj3 (diabete* or diabetic*)).tw. (14)

5 ((Non-insulin* or Non insulin* or Noninsulin*) adj3 depend* adj3 (diabete* or diabetic*)).tw. (261)

6 NIDDM.tw. (94)

7 T2D.tw. (194)

8 or/1-7 (6035)

9 depression.mp. (254593)

10 Affective Disorders/ (12354) Annotation: Include?

11 Dysthymic Disorder/ (1419)

12 (depress* or dysthym* or mood).ti. (102572)

13 (depression or depressive or depressed or dysthymic disorder* or mood disorder*).ti. (92547)

14 or/9-13 (268733)

15 8 and 14 (1010)

16 limit 15 to yr="2000 -Current" (956)

17 Client Education/ (3343)

18 Health Education/ (11089)

19 Health Promotion/ (19156)

20 Social Support/ (29831)

21 Self-Care Skills/ (3729)

22 INTERVENTION/ (46848)

23 exp PSYCHOTHERAPY/ (192350)

24 *COUNSELING/ (17264)

25 Treatment Guidelines/ (5292)

26 (psychoeducation or teach* or knowledge or inform).ti. (110843)

27 (program* or promot* or approach or strateg* or prevent* or intervention* or educat*).tw. (1351000)

28 (self-care or self-management or self-monitor* or self-help).tw. (26053)

29 ((health or patient or self) adj2 (knowledge or promotion or teach* or instruct* or learn* or information or coach*)).tw. (47693)

30 (treat* or manag* or recommendation* or guideline* or consensus or cpg* or pathway* or standard).tw. (1008747)

Annotation: treatment / too many drug treatment?

31 (cognitive or behavio?r or therapy or coach*).ti. (279847)

32 (psychotherapy or psycho-therapy or cbt or counseling).tw. (163714)

33 ((cognitive behavio?ral or cognitive or behavio?r*) adj3 (therap* or treat*)).tw. (62378)

34 ((social or family or peer) adj3 support).tw. (53796)

35 ((depression or mood or depressive) adj3 (treat* or therapy)).tw. (27377)

36 or/17-35 (2206941)

37 16 and 36 (753)

Search strategy **Cochrane**

Database: EBM Reviews

--------------------------------------------------------------------------------

1 Diabetes Mellitus, Type 2/ (9255)

2 (Type* adj3 ("2" or "II" or two*) adj3 (diabete* or diabetic*)).tw. (14669)

3 (adult* onset* adj3 (diabete* or diabetic*)).tw. (42)

Annotation: (adult onset* adj3 (diabete* or diabetic*)).tw.

4 ((Ketosis-resistant* or stable*) adj3 (diabete* or diabetic*)).tw. (187)

5 ((Non-insulin* or Non insulin* or Noninsulin*) adj3 depend* adj3 (diabete* or diabetic*)).tw. (1487)

6 NIDDM.tw. (982)

7 T2D.tw. (633)

8 or/1-7 (17335)

9 Depression/ (5773)

10 Depressive Disorder/ (4366)

11 Mood Disorders/ (454)

12 depressive disorder, major/ or dysthymic disorder/ (2614)

13 (depress* or dysthym* or mood).ti. (20017)

14 (depression* or depressive* or depressed or dysthymic disorder* or mood disorder*).tw. (40138)

15 or/9-14 (43162)

16 8 and 15 (489)

17 limit 16 to yr="2000 -Current" (468)

18 Patient Education as Topic/ (6614)

19 Health Education/ (2957)

20 Health Promotion/ (3802)

21 Self Care/ (2877)

22 exp Psychotherapy/ (16247)

23 Counseling/ (2953)

24 Social Support/ (2466)

25 Practice Guideline/ or Guideline/ (0)

26 (psychoeducation* or teach* or knowledge or inform*).ti. (6877)

27 (program* or promot* or approach or strateg* or prevent* or intervention* or educat*).tw. (234967)

28 (self-care or self-management or self-monitor* or self-help).tw. (7070)

Annotation: self-monitor*

29 ((health or patient or self) adj2 (knowledge or promotion or teach* or instruct* or learn* or information or coaching)).tw. (7047)

30 (treat* or manag* or recommendation* or guideline* or consensus or cpg* or pathway* or standard*).ti. (177507)

31 (cognitive or behavio?r* or therapy or coach*).ti. (81153)

32 (psychotherapy or psycho-therapy or cbt or counseling).tw. (11785)

33 ((cognitive behavio?ral or cognitive or behavio?r*) adj3 (therap* or treat*)).tw. (13534)

34 ((social or family or peer) adj3 support).tw. (3333)

35 ((depression or mood or depressive) adj3 (treat* or therapy)).tw. (10523)

36 or/18-35 (434694)

37 17 and 36 (412)
